# Amplicon-Based Profiling of Fungal Communities Associated with Scots Pine Bark Beetles: Selective Antagonism and Monoterpene Tolerance

**DOI:** 10.3390/ijms27104526

**Published:** 2026-05-18

**Authors:** Arunabha Khara, Sandipan Banerjee, Amrita Chakraborty, Jakub Dušek, Jiří Synek, Amit Roy

**Affiliations:** Faculty of Forestry and Wood Sciences, Czech University of Life Sciences Prague, Kamýcká 129, 165 00 Praha, Czech Republic; khara@fld.czu.cz (A.K.); banerjee@fld.czu.cz (S.B.); dusekjakub@fld.czu.cz (J.D.); synekj@fld.czu.cz (J.S.)

**Keywords:** *Ips sexdentatus*, *Ips acuminatus*, fungal community, ITS2 amplicon sequencing, monoterpenes, scanning electron microscopy, enzyme assay

## Abstract

Bark beetle–fungus associations are essential for nutrition, detoxification, and host colonisation, but their composition and function vary across developmental stages and environmental contexts. Hence, we characterised the fungal communities associated with two pine-feeding bark beetles, *Ips sexdentatus* (ISX) and *Ips acuminatus* (IAC), across developmental stages and compared wild-collected and laboratory-bred populations using ITS2 amplicon sequencing. Both beetle species maintained a stable core mycobiome dominated by *Kuraishia*, *Ogataea*, *Ophiostoma*, *Graphilbum*, and *Cyberlindnera*. These taxa have been earlier reported to be associated with nutrient provisioning, detoxification of host secondary metabolites, and chemical signalling. Adult beetles showed species-specific community differences, whereas wild-collected beetles, particularly IAC, harboured higher fungal diversity than laboratory populations, indicating a strong environmental effect. Beetles shared more fungal taxa with control wood than with gallery wood, suggesting possible fungal acquisition during feeding and concurrent restructuring of the wood mycobiome during infestation. Monoterpene bioassays with selected yeast symbionts showed differential growth responses to α-pinene, 3-carene, and terpinolene, and their mixture, with the mixture producing stronger inhibition than individual compounds. These yeast symbionts further displayed selective antagonistic activity in vitro against selected filamentous fungi, including entomopathogenic taxa, along with detectable lytic and digestive enzyme activities. Together, our findings highlight a link between community structure, predicted functions, and observed interaction phenotypes, providing a strong basis for future mechanistic studies of beetle–fungus–conifer interactions.

## 1. Introduction

Bark beetles (Coleoptera: Curculionidae: Scolytinae) are among the most consequential forest pests, capable of triggering landscape-scale outbreaks and substantial economic losses [1]. In Europe, climate change, combined with human-driven disturbances, has amplified the aggressiveness of pine-feeding species such as *Ips acuminatus* and *I. sexdentatus*, which now frequently colonise not only weakened but also seemingly healthy trees [2,3,4,5,6]. For instance, in the Czech Republic alone, bark beetle infestations on pinewood increased from ~10,000 m^3^ in 2009 to nearly 80,000 m^3^ in 2019 [7]. Climate change has exacerbated bark beetle outbreaks by weakening tree defences through rising temperatures, altered precipitation, and frequent drought and heat events [8,9]. Drought, in particular, reduces host tree vigour, enabling beetle populations to surpass epidemic thresholds [10].

Successful colonisation of pine hosts requires beetles to overcome robust constitutive and induced defences. Physical barriers (lignin and suberin-rich tissues) and chemical defences (phenolics and terpenoids) impede invasion and development [11]. Bark beetle–fungus mutualism is one such crucial interaction in which fungal partners have evolved specific adaptations that enable beetles to thrive in the toxin-laden conifer environment and utilise the nutrient-deficient phloem [12]. These fungal associates can include filamentous fungi, yeasts, and other taxa occurring on beetle body surfaces, in galleries, or within internal microhabitats (i.e., gut, hemolymph, or any other specialised tissue) and have been linked to potential ecological functions such as nutrient acquisition, degradation or tolerance of host defence compounds, modification of volatile compounds, and interactions with other microbes [13]. For instance, in ambrosia beetles, the fungal symbionts reside in specialised structures (i.e., mycangia), whereas in *Ips* beetles, fungi produce spores that can easily attach to beetle bodies [14,15]. These spores contain a well-protected sheath that prevents digestion inside the beetle gut [16]. These fungal associates contribute to beetle fitness through multiple, complementary functions, including nutrient acquisition, detoxification of plant defence compounds, and the production of volatile compounds to facilitate beetle communication [17]. For example, fungal partners degrade the complex biopolymers of wood, such as hemicellulose, lignin, and cellulose, making them nutritionally accessible to their host beetles [18,19]. However, these roles are often context-dependent and require direct functional validation, particularly when inferred from community composition or in vitro assays.

Filamentous fungi associated with bark beetles have received considerable attention because of their frequent occurrence in beetle galleries and their potential roles in host colonisation and gallery ecology [12]. For example, several fungal associates of the Eurasian spruce bark beetle (e.g., *Ceratocystis polonica*, *Grosmannia europhioides*, and *Grosmannia penicillate*) have been linked to the degradation of spruce phenolic compounds, which may facilitate host colonisation [20,21]. Other bark beetle-associated fungi, such as *Grosmannia*, *Ophiostoma*, and *Endoconidiophora*, can metabolise host-derived monoterpenes or transform beetle-associated volatiles, potentially influencing aggregation and competition dynamics [22,23,24]. Fungal mutualists also provide bark beetles with protection against other pathogens. Some fungal associates may also inhibit entomopathogenic fungi; for example, *Leptographium abietinum* has been shown to inhibit *Beauveria bassiana* [25]. In addition to filamentous fungi, yeasts are increasingly recognised as important members of bark beetle-associated mycobiomes [26]. Several reports documented recurrent associations with yeasts, including *Kuraishia*, *Ogataea*, *Candida*, and related taxa, suggesting that yeasts are not merely incidental contaminants but persistent members of bark beetle microbial communities [26,27]. These yeasts may contribute to digestion and nutritional supplementation in nutrient-poor phloem, tolerance or transformation of host-derived secondary metabolites, and modulation of volatile compounds involved in beetle chemical ecology [26]. Therefore, it is evident that fungal symbionts, including yeasts, play a crucial role in the survival of bark beetles under challenging environments [28]. However, many of these proposed roles remain context-dependent and require direct functional validation in vivo.

Despite extensive work on symbioses in several model bark beetles, including the red turpentine and mountain pine beetles [29,30] and Eurasian spruce bark beetle [31,32], comparatively little is known about the mycobiomes of the pine-feeding *Ips* species *I. acuminatus* and *I. sexdentatus*, particularly across ontogeny. These two species are sympatric bark beetles associated with Scots pine (*Pinus sylvestris*) and may exploit overlapping host resources. However, they can differ in colonisation niche, population dynamics, and ecological impact, making them an interesting model for comparing how closely related pine-feeding beetles shape their fungal communities [33]. Furthermore, the understanding of the fungal communities across potential host–environment filters, such as laboratory versus wild conditions and the surrounding wood mycobiome, remains insufficiently studied for these taxa. The surrounding host environment is also likely to influence beetle-associated fungal assemblages. Scots pine phloem and resin contain chemically active monoterpenes that can affect both insects and their microbial associates [22,34,35]. Testing beetle-associated fungal responses to these compounds provides a targeted way to assess how they tolerate or respond to host-related chemical conditions. In addition to host chemical defences, bark beetles are exposed to microbial antagonists, including entomopathogenic fungi such as *Beauveria* spp. [36]. These exposures may create selection for microbial associates that influence pathogen establishment or fungal community structure within beetle galleries. Therefore, examining such interactions can generate testable hypotheses about the possible ecological roles of fungal symbionts in bark beetle systems.

In our study, we characterised the fungal communities associated with *I. acuminatus* and *I. sexdentatus* across different life stages using high-throughput fungal ITS2 amplicon sequencing. We also compared lab-bred and wild-collected adults to evaluate environmental contributions to fungal assemblages. To complement the community analysis, we tested selected culturable beetle-associated yeasts for their responses to ecologically relevant monoterpenes—α-pinene, 3-carene, and terpinolene—and their mixture, because they represent major and ecologically relevant components of Scots pine (*Pinus sylvestris*) resin [34] and are therefore among the compounds likely encountered by beetle-associated fungi during host colonisation. The present study addressed three testable questions: (i) how fungal community composition varies between beetle species, developmental stages, and rearing environments; (ii) how beetle-associated mycobiomes relate to the surrounding wood mycobiome; and (iii) how selected yeast symbionts respond in vitro to host-associated monoterpenes and interact with selected filamentous fungi, including entomopathogenic taxa. We hypothesise that both *Ips* species harbour a conserved core mycobiome across developmental stages while also showing stage- and environment-dependent variation. We further expected wild-collected adults to harbour more diverse and wood-influenced fungal assemblages than laboratory-bred adults. Finally, we hypothesised that monoterpene exposure would differentially affect beetle-associated yeast growth, with mixtures potentially producing stronger inhibitory effects than individual compounds, and that gut-associated yeast symbionts may contribute to potential antagonism toward entomopathogenic fungi and possess complementary enzyme activities that could enhance it. By integrating community profiling with targeted interaction and enzymatic assays of selected yeast symbionts, we relate beetle mycobiome structure to putative ecological roles in nutrition, detoxification, and chemical signalling, and we generate a set of testable hypotheses for future evolutionary and functional evaluation.

## 2. Results

### 2.1. Sequencing Statistics

The culture-independent high-throughput Illumina paired-end sequencing of life stages (larval, pupal, and adult), different populations (lab-bred and wild collection) of the two *Ips* pine beetles, *I. sexdentatus* (ISX) and *I. acuminatus* (IAC), and wood tissue (control wood and fed/gallery wood) samples generated 8,670,808 raw reads. A Phred Quality score > 30 was used for quality checks to obtain 7,147,099 clean reads (File S1). The rarefaction curve and Good’s coverage indicator (>99%) indicated the sequencing depth for all samples (Table S1, Figure S1). The sample descriptions, including the abbreviations used, are provided in Table 1.

### 2.2. Mycobiome Composition and Diversity

#### 2.2.1. Fungal Taxonomic Abundance

**Figure 1:**
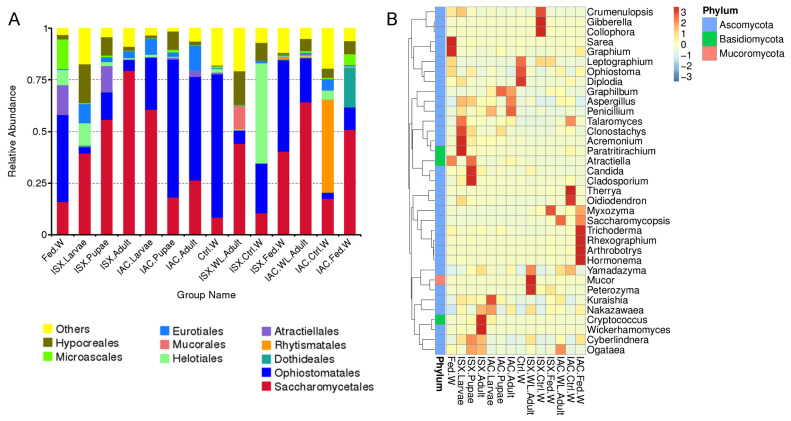
Fungal diversity in life stages (lab-bred), wild-collected adult pine-feeding beetles, and wood samples. (**A**) Bar diagram depicting beetle fungal community relative abundance at the order level (top 10). (**B**) Thirty-five dominant fungal genera among life stages (lab-bred), wild adult, and wood samples are represented in the heatmap. The colour gradient represents the relative abundance of ASVs, with lighter colours indicating lower abundance and darker colours indicating higher abundance for a specific fungal genus. IAC—*I. acuminatus*; ISX—*I. sexdentatus*; WL—wild-collected; Ctrl. W—Control uninfested wood; Fed. W—Gallery wood.

The fungal ITS2 sequences were categorised into 2506 ASVs, representing 63 fungal orders (File S2). The computed Goods coverage index (0.98), representing filtered sequence reads (*n* > 5), validated that most ASVs in the samples were detected, with about 2% remaining undetected during sequencing. The predominant fungal orders were Saccharomycetales (ISX.Larvae—0.39 ± 0.08, ISX.Pupae—0.55 ± 0.17, ISX.Adult—0.79 ± 0.06, IAC.Larvae—0.60 ± 0.04, ISX.WL.Adult—0.44 ± 0.06, IAC.WL.Adult—0.64 ± 0.07, IAC.Fed.W—0.51 ± 0.16) and Ophiostomatales (IAC.Pupae—0.67 ± 0.07, IAC.Adult—0.50 ± 0.12, Fed.W—0.42 ± 0.12, Ctrl.W—0.69 ± 0.04, ISX.Fed.W—0.44 ± 0.01) for all beetle and wood samples except ISX.Ctrl.W, where Helotiales (0.48 ± 0.03) was the dominant order (Figure 1A, File S3). The heatmap depicting the top 35 fungal genera in the different life stages of both beetles showed varied dominance of fungal genera at specific life stages (Figure 1B). For instance, in the case of ISX beetle life stages, the larvae were dominated by the genera *Talaromyces*, *Clonostachys*, *Acremonium*, and *Paratritirachium*. While *Atractiella*, *Candida*, and *Cladosporium* were the predominant fungal genera in pupae, *Cryptococcus* and *Wickerhamomyces* were prevalent in adults. Similarly, in IAC life stages, an abundance of *Kuraishia* and *Nakazawaea* was observed in IAC larvae, while *Graphilbum* and *Aspergillus* were the dominant species in adults.

#### 2.2.2. ASV Abundance

**Figure 2:**
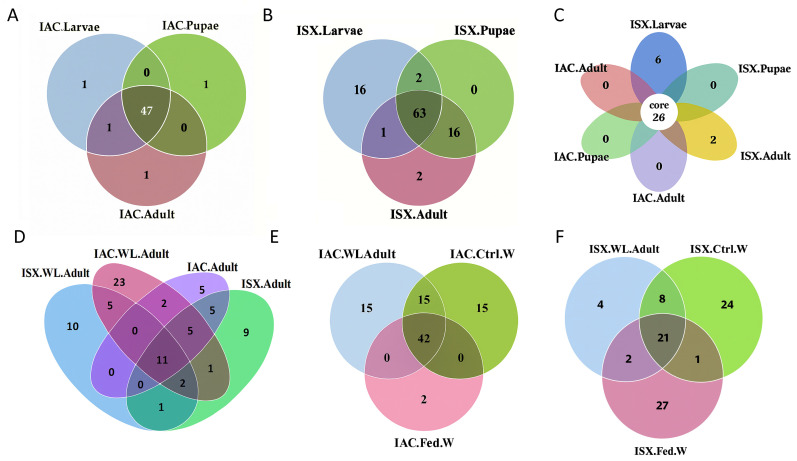
Mycobiome composition in life stages (lab-bred), wild adult pine-feeding beetles, and wood samples. (**A**) Venn diagram representing fungal ASV distribution in *I. acuminatus* life stages (IAC.Larvae, IAC.Pupae, and IAC.Adult). (**B**) Venn diagram illustrating fungal ASV distribution in *I. sexdentatus* life stages (ISX.Larvae, ISX.Pupae, and ISX.Adult). (**C**) The core ASVs across the life stages of the two pine beetles are depicted by a flower diagram. (**D**) The shared and unique ASVs present in *I. acuminatus* and *I. sexdentatus* wild beetles (IAC.WL.Adult and ISX.WL.Adult) and lab-bred beetles (IAC.Adult and ISX.Adult) are represented in the Venn diagram. (**E**) The fungal ASV contribution of the wood mycobiome in shaping the *I. acuminatus* mycobiome is shown in the Venn diagram. (**F**) The fungal ASV contribution of the wood mycobiome in shaping the *I. sexdentatus* mycobiome is shown in the Venn diagram.

The ASV analysis revealed a diverse distribution of ASVs across different developmental stages for both *Ips* beetles. In *I. acuminatus* (IAC), the core fungal communities across life stages comprised 47 ASVs, categorised into 16 families and 20 genera, with the predominance of families Aspergillaceae, Ophiostomataceae, Trichocomaceae, and Saccharomycetales_fam_Incertae_sedis and fungal genera including *Penicillium*, *Graphilbum*, *Ophiostoma*, *Talaromyces*, and *Kuraishia* (Figure 2A, File S4). However, it is essential to note that each ASV may not represent a distinct species.

The core mycobiome shared between *I. sexdentatus* (ISX) life stages was represented by 63 ASVs, which were differentiated into 26 families and 32 genera (Figure 2B, File S5). Highly abundant fungal families included Saccharomycetales_fam_Incertae_sedis, Pichiaceae, and Ophiostomataceae and fungal genera such as *Kuraishia*, *Ogataea*, *Ophiostoma*, and *Graphilbum* were prevalent. The ASV abundance data also revealed the core fungal mycobiome, including all life stages (larvae, pupae, and adults) of both *Ips* pine beetles, which accounted for 26 ASVs categorised into 14 families and 16 genera (Figure 2C, File S6). The core mycobiome comprised highly abundant fungal genera, including *Nakazawaea*, *Ogataea*, *Ophiostoma*, *Graphilbum*, *Kuraishia*, and *Talaromyces*. This pattern, in which the number of shared ASVs is higher than individual stage-specific ASV abundance, suggests a conserved mycobiome structure across developmental stages within each species, with a stable set of core fungal taxa persisting throughout host development, despite stage-specific shifts in abundance.

When comparing the lab-bred and wild-collected beetles in both *Ips* species, the number of unique ASVs was higher than the number of shared ones, indicating that environmental factors influence the shaping of the mycobiome composition (Figure 2D, File S7). In IAC wild-collected beetles, the unique ASVs comprised 4 families and 5 genera (23 ASVs), whereas in IAC lab-bred adults, they included 2 families and 2 genera (5 unique ASVs). Similarly, for ISX, wild-collected and lab-bred beetles had a higher number of unique ASVs (10 and 9, respectively) compared to one shared ASV. The unique ASVs in wild-collected beetles belonged to 7 families and 7 genera, whereas in lab-bred beetles, they belonged to 4 families and 4 genera. The wild beetles documented more unique ASVs and greater diversity than the lab-bred adults, indicating that the fungal communities associated with beetles under natural conditions were more diverse than those under laboratory breeding. However, such interpretations should be viewed cautiously, as the patterns are based on a limited sample set from a single geographic region. Notably, in both beetle species, the ASV shared between wild-collected and laboratory-bred individuals belonged to the genus *Ophiostoma*, suggesting a conserved, and potentially important, symbiotic association that persists across rearing conditions in the populations examined here.

For both beetle species, wild-collected beetles shared more ASVs with control wood samples (IAC.WL.Adult and IAC.Ctrl.W—15 ASVs, ISX.WL.Adult and ISX.Ctrl.W—8ASVs) rather than with fed (gallery) wood samples (IAC.WL.Adult and IAC.Fed.W—0 ASVs, ISX.WL.Adult and ISX.Fed.W—2 ASVs). Subsequently, IAC control and fed wood had 15 and 2 unique ASVs, respectively. Similarly, ISX control and fed wood samples had respective 24 and 27 unique ASVs (Figure 2E,F, File S8). These patterns suggest that beetle feeding induces a marked shift in the wood mycobiome.

#### 2.2.3. Alpha Diversity

**Figure 3:**
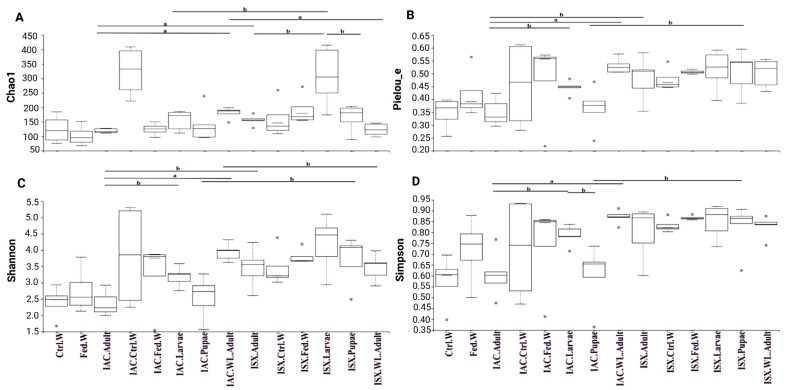
Alpha diversity indices depicted as boxplots for lab-bred, wild-collected *Ips* pine beetles and their wood tissue. (**A**) Fungal richness estimation (Chao1) depicts significant difference among different life stages, wild-collected adults, and wood samples. (**B**) Fungal evenness estimation by the Pielou index. (**C**) Shannon and (**D**) Simpson indexes illustrate fungal diversity. Statistically significant differences among various groups at *p* < 0.05 (designated as “b”), *p* < 0.01 (designated as “a”) were observed using the Kruskal–Wallis pairwise group test. IAC—*I. acuminatus*; ISX—*I. sexdentatus*; WL—wild-collected; Ctrl. W—Control uninfested wood; Fed. W—Gallery wood.

The fungal mycobiome diversity was studied using alpha diversity indices (community richness, evenness, and diversity), revealing significantly different developmental stage-specific fungal associations. In *I. acuminatus* developmental stages, all of the alpha diversity indices were higher in IAC.Larvae than in IAC.Pupae and IAC.Adult samples (Figure 3, Table S1, File S9).

IAC.Larvae (Pielou—0.45 ± 0.06, Shannon—3.19 ± 0.14, Simpson—0.79 ± 0.02) and IAC.Adult samples (Pielou—0.35 ± 0.02, Shannon—2.37 ± 0.17, Simpson—0.61 ± 0.05) exhibited significant differences (*p* < 0.05) in fungal evenness and diversity. Similar to *I. acuminatus* life stages, in *I. sexdentatus* developmental stages, the fungal alpha diversity indices (community richness, evenness, and diversity) were higher in ISX.Larvae than in ISX.Pupae and ISX.Adult samples (Figure 3, Table S1, File S9). ISX.Larvae (Chao1: 308.84 ± 45.22) samples showed significant differences (*p* < 0.05) in community richness compared to the respective ISX.Pupae (Chao1—164.52 ± 20.98) and ISX.Adult (Chao1—155.95 ± 8.1) samples. Similarly, when comparing the larval samples of both species (IAC and ISX), significant differences (*p* < 0.05) in fungal richness were observed. The adult samples for both species also showed significant differences (*p* < 0.01) in fungal richness and evenness. However, the pupal stage did not show significant differences in community richness and evenness. ISX.Pupae (Shannon—3.7 ± 0.33, Simpson—0.6 ± 0.06) revealed significantly higher fungal diversity than IAC.Pupae (Shannon—2.55 ± 0.29, Simpson—0.82 ± 0.05) (*p* < 0.05).

All of the alpha diversity indices showed significant differences (*p* < 0.01) between wild-collected and lab-bred *I. acuminatus* beetles. However, such significant differences were not observed between wild and lab-bred *I. sexdentatus* beetles. By contrast, studying the wood mycobiome in the IAC.Ctrl.W (324.13 ± 45.29) samples showed significantly (*p* < 0.05) higher fungal richness than their respective fed wood samples (124.69 ± 11.07). Moreover, the fungal evenness and Shannon diversity index were higher in IAC.Ctrl.W samples, but the Simpson diversity index was higher in IAC.Fed.W samples. However, all of the alpha diversity indices were higher in ISX.Fed.W samples compared to its control wood samples. Such observations suggest that beetle feeding affects the wood microbiome, and vice versa.

#### 2.2.4. Beta Diversity

**Figure 4:**
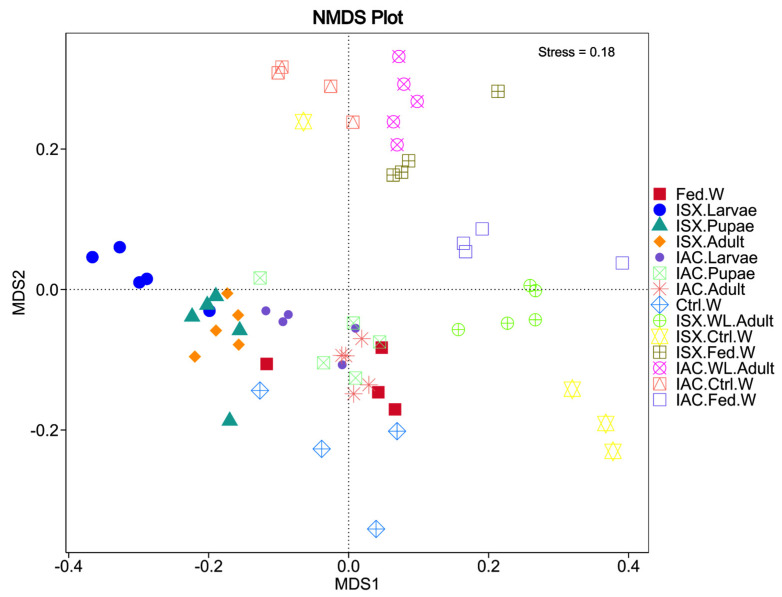
Beta diversity analysis. Non-metric multidimensional scaling (NMDS) represents the fungal diversity variation among life stages and wild *Ips* pine beetles. IAC—*I. acuminatus*; ISX—*I. sexdentatus*; WL—wild-collected; Ctrl. W—Control uninfested wood; Fed. W—Gallery wood.

The NMDS and PCoA analysis based on the unweighted and weighted UniFrac distances, respectively (Figure 4 and Figure S2), revealed clear separation of life stages.

Notably, the fungal communities in the larvae of IAC and ISX exhibited significant differences both within and between groups (Tables S2 and S3). Furthermore, Metastat analysis indicated genus-specific shifts across the life stages of the pine beetles. Within *I. acuminatus*, larvae (IAC.Larvae) were enriched with *Kuraishia*, whereas adults (IAC.Adult) and pupae (IAC.Pupae) were dominated by *Graphilbum* (Table 2). A comparable pattern emerged in *I. sexdentatus* (ISX): *Talaromyces* predominated in larvae (ISX.Larvae), while *Ogataea* and *Cryptococcus* were more abundant in pupae and adults (ISX.Pupae and ISX.Adult). Wild-collected versus laboratory-bred contrasts further underscored these differences.

In *I. acuminatus*, wild-collected beetles harboured significantly higher levels of *Kuraishia*, *Ogataea*, and *Ophiostoma*, whereas lab-bred beetles were enriched with *Graphilbum* and *Atractiella*. In *I. sexdentatus*, wild-collected beetles were characterised by significantly greater abundances of *Leptographium*, *Peterozyma*, and *Myxozyma*, while laboratory cohorts showed prevalences of *Kuraishia*, *Ogataea*, and *Wickerhamomyces*. Consistent presence was also evident in wood samples: IAC.Ctrl.W exhibited significantly higher abundances of *Therrya*, *Oidiodendron*, and *Talaromyces*, in contrast to IAC.Fed.W, where *Cyberlindnera*, *Ophiostoma*, and *Saccharomycopsis* predominated. In the ISX wood samples, ISX.Ctrl.W was dominated by *Crumenulopsis* and *Gibberella*, whereas *Graphilbum* and *Kuraishia* were abundant in ISX.Fed.W. Finally, multivariate testing (ADONIS and ANOSIM) confirmed significant compositional differences among life stages in both beetle species (Tables S2 and S3).

**Figure 5:**
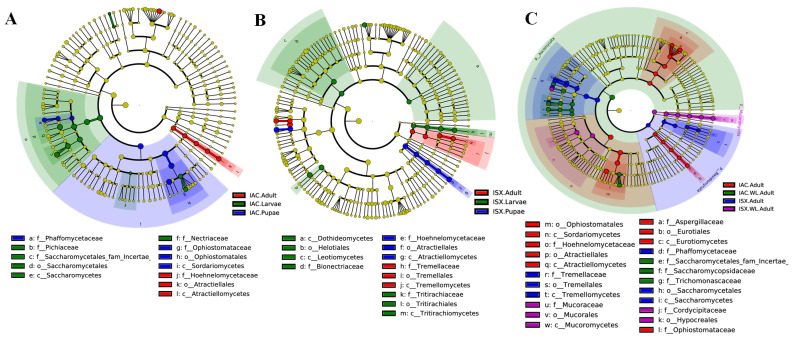
LefSe analysis. (**A**) Cladogram depicting significantly different fungal biomarkers across *I. acuminatus* life stages. (**B**) Cladogram representing significantly distinct fungal biomarkers across *I. sexdentatus* life stages. (**C**) Cladogram depicting significant fungal biomarkers between wild-collected and lab-bred pine-feeding beetles. Taxonomic levels (phylum to genus) are outlined in the circle from inward to outward. The relative abundance of any taxon is portrayed by the size of the circles. Different coloured circles represent different life stages of the pine beetles. The letters above the different coloured circles indicate a specific fungal biomarker. The yellowish-green circles illustrate non-significant fungal species. IAC—*I. acuminatus*; ISX—*I. sexdentatus*; WL—wild-collected.

Our study further investigated the presence of significantly dominant fungal genera across different life stages using LefSe analysis (Figure 5, Figures S3 and S4). In the case of *I. acuminatus*, distinct fungal families were identified as biomarkers at various life stages: the family Hoehnelomycetaceae (order: Tremellales) was prevalent in the IAC.Adult stage, while Ophiostomataceae (order: Ophiostomatales) was a key biomarker in IAC.Pupae. Additionally, Pichiaceae and Nectriaceae (order: Saccharomycetales) were dominant in IAC.Larvae (Figure 5A and Figure S4A). Similarly, in *I. sexdentatus*, biomarkers varied across developmental stages: ISX.Larvae exhibited fungal biomarkers from the orders Helotiales and Tritirachiales, with families Bionectriaceae and Tritirachiaceae, while ISX.Pupae contained biomarkers from the family Hoehnelomycetaceae (order: Tremellales), and ISX.Adult was characterised by biomarkers from the family Tremellaceae (order: Tremellales) (Figure 5B and Figure S4B).

When comparing lab-bred versus wild-collected beetles, distinct fungal biomarkers were observed in *I. acuminatus*: wild-collected beetles showed biomarkers from the families Saccharomycetales_fam_Incertae and Saccharomycopsideceae, whereas lab-bred adults harboured biomarkers from the families Phaffomycetaceae and Tremellaceae (orders: Saccharomycetales and Tremellales) (Figure 5C and Figure S4C). Similarly, in *I. sexdentatus*, lab-bred beetles showed a dominance of families Phaffomycetaceae and Tremellaceae (orders: Saccharomycetales and Tremellales), while wild-collected specimens featured the family Mucoraceae (order: Mucorales) (Figure 5C and Figure S4C).

Furthermore, distinct fungal biomarkers were identified across wild-collected beetles, control wood, and fed wood samples (Figures S3 and S4). In IAC.WL.Adult, the biomarkers included the families Cordycipitaceae and Ophiostomataceae, while ISX.WL.Adult samples showed biomarkers from the families Phaffomycetaceae, Cordycipitaceae, and Mucoraceae. For *I. acuminatus* wood samples, control wood contained the families Trichocomaceae, Myxotrichaceae, and Rhytismataceae, whereas fed wood exhibited biomarkers from the families Dothioraceae and Hypocreaceae. In *I. sexdentatus*, control wood (ISX.Ctrl.W) harboured Helotiales_fam_Incertae, while fed wood (ISX.Fed.W) showed Saccharomycetales_fam_Incertae as the dominant fungal biomarker.

### 2.3. Functional Prediction of Beetle Mycobiome

**Figure 6:**
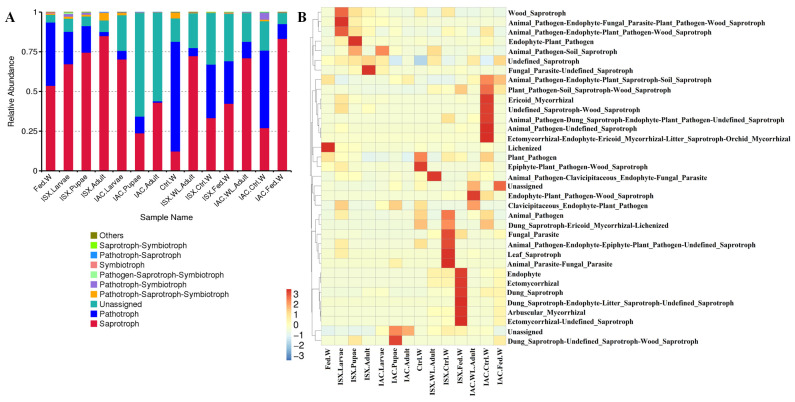
FUNGuild-based prediction of different functional groups (guilds) in the fungal community. (**A**) Bar diagram revealing the relative abundance of different fungal functional groups at the mode level. (**B**) Heatmap representing different fungal functional groups at the guild level. IAC—*I. acuminatus*; ISX—*I. sexdentatus*; WL—wild-collected; Ctrl. W—Control uninfested wood; Fed. W—Gallery wood.

The putative roles of fungal communities across various life stages of both *Ips* species were predicted using FUNGuild (Figure 6). Both pine-feeding beetle species exhibited a prominent presence of saprotrophs, with notable variations in abundance, except for the pupal and adult stages of *I. acuminatus*. In the life stages of *I. sexdentatus*, saprotrophs were classified as wood saprotrophs, soil saprotrophs, and undefined saprotrophs. Notably, wild adults displayed a high prevalence of saprotrophs (72%), alongside a marked abundance of animal pathogens. Similarly, in *I. acuminatus*, the larvae (IAC.Larvae), pupae (IAC.Pupae), and wood-living adults (IAC.WL.Adult) exhibited substantial presences of soil and wood saprotrophs.

### 2.4. Fungal Relative Abundance Determined by Quantitative PCR Assay

Quantitative PCR (qPCR) analysis was conducted for all developmental stages (larvae, pupae, and adults) of *I. sexdentatus*. For *I. acuminatus*, qPCR analysis was limited to adult samples because sufficient material from other developmental stages was unavailable. Therefore, qPCR-based comparisons across developmental stages were possible only for *I. sexdentatus*, while *I. acuminatus* results should be interpreted as adult-specific validation.

#### 2.4.1. *Ips sexdentatus* Life Stages

**Figure 7:**
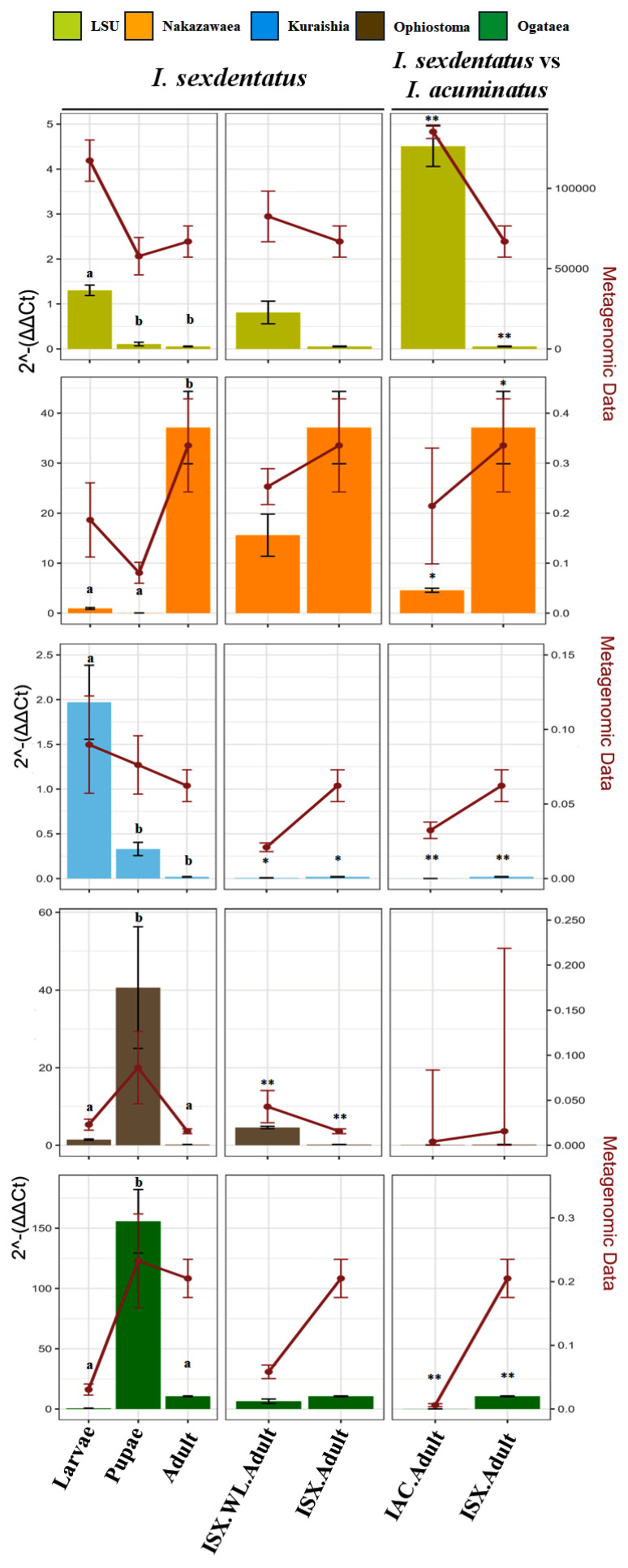
Quantitative PCR (qPCR) assays were performed to estimate the relative abundances of the predefined fungal taxa in the two pine-feeding beetle species. Fold change in fungal abundance was estimated using the 2^−ΔΔCt^ method normalised against stable reference genes (ISX: β-Tubulin; IAC: EF1α and RPL7). Values are presented as means ± SE from beetle samples (*n* = 4). Statistical contrasts were determined using ANOVA, with post-hoc tests and *t*-tests used for pairwise comparisons. Different letters indicate differences between groups (*p* < 0.01). Full statistical results are presented in File S10. IAC—*I. acuminatus*; ISX—*I. sexdentatus*; WL—wild-collected. *p* < 0.05 (designated as “*”), *p* < 0.01 (designated as “**”).

qPCR analysis revealed significant abundance differences in the total fungal population as well as selected fungal genera across life stages in *I. sexdentatus*, which corroborated the metagenomic sequencing results (Figure 7, File S10). The total fungal population in larvae was significantly higher than that in all other life stages (*p* < 0.01), while pupae and adults did not differ significantly. A similar pattern was observed for *Kuraishia*, where higher abundance was observed in larvae compared to adults and pupae (*p* < 0.01). For *Ophiostoma* and *Ogatea*, the pupae life-stage exhibited significantly higher abundance than adults (*p* < 0.05, *p* < 0.01) and larvae (*p* < 0.05, *p* < 0.01). However, for *Nakazawaea*, adults exhibited significantly higher abundance than larvae and pupae (*p* < 0.01 for both).

#### 2.4.2. Wild-Collected Adults vs. Lab-Bred Adults

Comparing wild-collected and lab-bred adults revealed significant differences in the abundances of *Kuraishia* (*p* < 0.05) and *Ophiostoma* (*p* < 0.01) (Figure 7, File S10). However, no such significant differences were observed for other genus-specific primers.

#### 2.4.3. *I. sexdentatus* vs. *I. acuminatus* Adults

Comparisons between ISX and IAC adult beetles showed significant differences for all primers except *Ophiostoma* (Figure 7, File S10). The relative abundance of the total fungal population was markedly higher in IAC adults (*p* < 0.01). However, for *Nakazawaea* (*p* < 0.05), *Kuraishia* (*p* < 0.05), and *Ogatea* (*p* < 0.01), their relative abundances were significantly higher in ISX adults. These findings suggest that certain fungal associates are more prevalent in ISX populations, while others, such as *Ophiostoma*, are maintained at comparable levels regardless of geographic origin.

### 2.5. Effects of Monoterpenes on Pine Bark Beetle-Associated Yeasts

**Figure 8:**
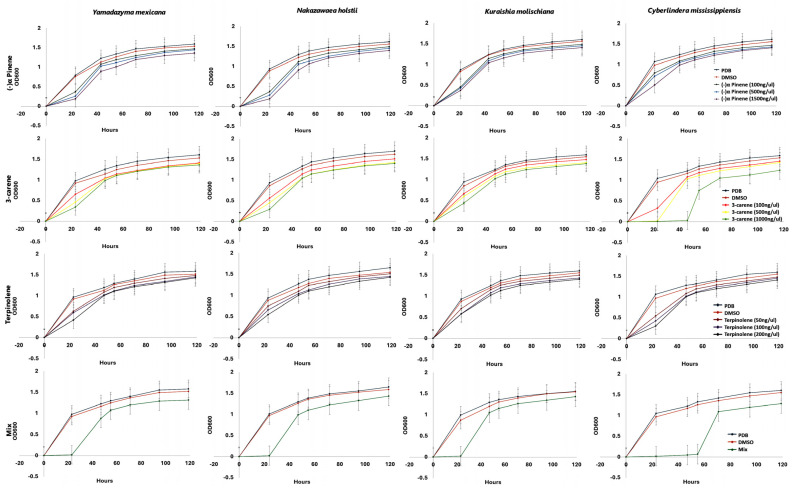
Bioassay of yeast isolates subjected to monoterpenes. Growth response of four yeast isolates (*Yamadazyma mexicana*, *Nakazawaea holstii*, *Kuraishia molischiana*, and *Cyberlindnera mississippiensis*) to different monoterpenes (α-pinene, 3-carene, and terpinolene) and their mixture.

The four yeasts associated with the pine bark beetles, *Y. mexicana*, *N. holstii*, *K. molischiana*, and *C. mississippiensis*, showed distinct growth responses to individual monoterpene treatment as well as to the monoterpene blend (Figure 8). In the control treatments, consisting of growth media (PDB) with no supplements and growth media with DMSO, all isolates exhibited consistent growth and a sharp rise in turbidity at approximately 40 h. Exposure to individual monoterpenes, including (−)-α-pinene, 3-carene, and terpinolene, caused species-specific growth reductions. The highest degree of growth inhibition due to monoterpenes was most frequently observed with (−)-α-pinene, particularly in *Y. mexicana* and *N. holstii*. By contrast, 3-carene and terpinolene caused moderate inhibition in most isolates. Notably, the growth curves indicated that inhibition was most pronounced during the first 20–24 h, followed by a later recovery or lower inhibition, which suggests a possible acclimation period. The monoterpene blend (a mix of α-pinene, 3-carene, and terpinolene) mimicking the more complex terpene state encountered within infested host tissue was consistently and significantly most effective at inhibiting growth in all the yeast species. It is important to note that the monoterpene assays evaluated the growth responses of selected yeast isolates following exposure to host-associated monoterpenes. Thus, these assays provide evidence of differential monoterpene tolerance or sensitivity but do not directly demonstrate detoxification. Confirming terpene detoxification or transformation would require chemical profiling, such as GC-MS, to quantify monoterpene depletion and identify potential degradation metabolites.

### 2.6. Gut Microbial Colonisation and Biofilm Formation

The symbiotic association in the different gut regions of *I. sexdentatus* was observed through an SEM study (Figure S5). Colonisation of yeast in specific gut regions, i.e., midgut (Figure S5A,B) and hindgut (Figure S5C,D), was very prominent and significant. Similarly, in vitro biofilm formation by the gut isolates was observed under SEM, indicating the adhesion of yeast cells to experimental surfaces. In addition, these gut isolates exhibited multi-layered, three-dimensional co-aggregation and a tendency to attach to solid surfaces (Figure S6).

### 2.7. Fungal Interactions In Vitro

**Figure 9:**
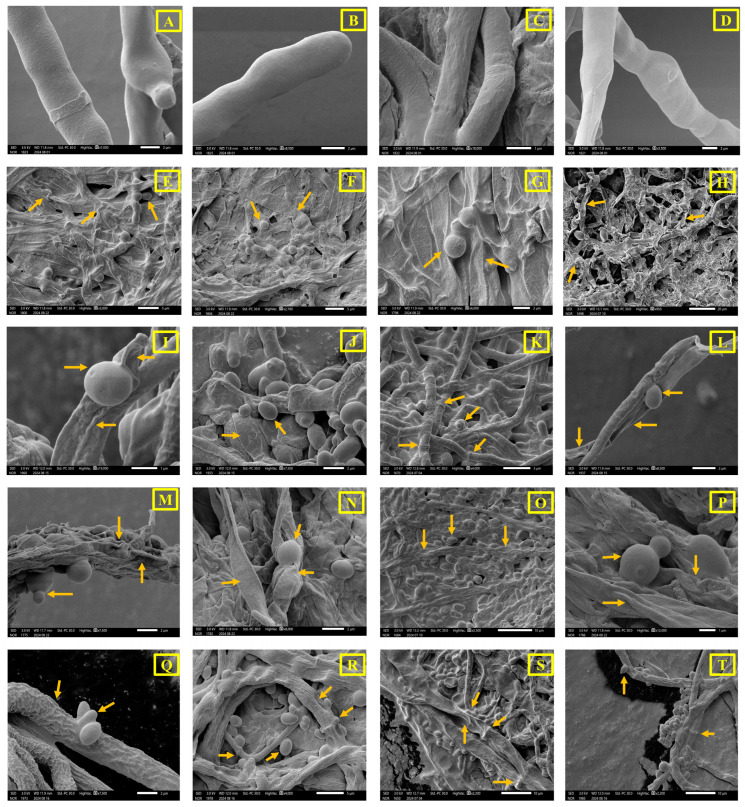
Interaction of symbiotic yeasts against entomopathogenic pathogenic fungi. (**A**–**D**) Healthy fungal mycelial structure of entomopathogenic fungi [(**A**) *Absidia caatinguens*, (**B**) *Beauvaria bassiana*, (**C**) *Clonostachys rosea*, and (**D**) *Trichoderma* sp.]. (**E**–**H**) SEM observations of entomopathogenic fungi *Absidia caatinguens* and yeasts isolates [(**E**) *N. holstii*, (**F**) *C. mississippiensis*, (**G**) *Y. mexicana*, and (**H**) *K. molischiana*]. (**I**–**L**) SEM observations of entomopathogenic fungi *Beauveria bassiana* and yeast isolates [(**I**) *N. holstii*, (**J**) *C. mississippiensis*, (**K**) *Y. mexicana*, and (**L**) *K. molischiana*]. (**M**–**P**) SEM observations of entomopathogenic fungi *Clonostachys rosea* and yeast isolates [(**M**) *N. holstii*, (**N**) *C. mississippiensis*, (**O**) *Y. mexicana*, and (**P**) *K. molischiana*]. (**Q**–**T**) SEM observations of entomopathogenic fungi *Absidia caatinguens* and symbiotic yeast [(**Q**) *N. holstii*, (**R**) *C. mississippiensis*, (**S**) *Y. mexicana*, and (**T**) *K. molischiana*]. Yellow arrows indicated different magnitudes of entomopathogenic fungal hyphae damage.

**Figure 10:**
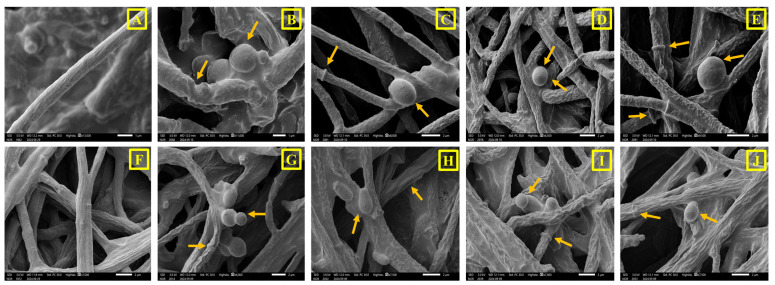
Interaction of symbiotic yeasts against non-entomopathogenic fungi. (**A**) Healthy fungal mycelial structure of non-entomopathogenic fungi *Ophiostoma hongxingense*. (**B**–**E**) SEM observations of non-entomopathogenic fungi *Ophiostoma hongxingense* and symbiotic yeast [(**B**) *N. holstii*, (**C**) *C. mississippiensis*, (**D**) *Y. mexicana*, and (**E**) *K. molischiana*]. (**F**) Healthy mycelial structure of *Ophiostoma piceae*. (**G**–**J**) SEM observations of non-entomopathogenic fungi *Ophiostoma multisynnematum* and yeast isolates [(**G**) *N. holstii*, (**H**) *C. mississippiensis*, (**I**) *Y. mexicana*, and (**J**) *K. molischiana*]. The arrows indicate little to no damage to non-entomopathogenic fungal hyphae.

The antifungal potential of the four yeast isolates was observed under SEM, where prominent degradation and deformation of the selected EPF and NEPF mycelial structures were visualised. The healthy mycelial structures of the EPF (Figure 9A–D) and NEPF (Figure 10A,F) and individual yeast cellular morphologies (Figure S7) were also documented through SEM. The antagonistic effect of all four gut symbionts on the EPF, e.g., *A. caatinguens* (Figure 9E–H), *B. bassiana* (Figure 9I–L), *C. rosea* (Figure 9M–P), and *Trichoderma* sp. (Figure 9Q–T), was reflected by the massive deconstruction of the EPF mycelial structure along with interactive yeast cells, specifically the dismantled mycelial morphology, in comparison to healthy fungal mycelia. Interestingly, the yeast symbionts were unable to prominently disorient the NEPF mycelial structures of *Ophiostomatoid hongxingense* (Figure 10B–E) and *Ophiostomatoid piceae* (Figure 10G–J) in comparison to EPF.

### 2.8. Enzyme Production

**Figure 11:**
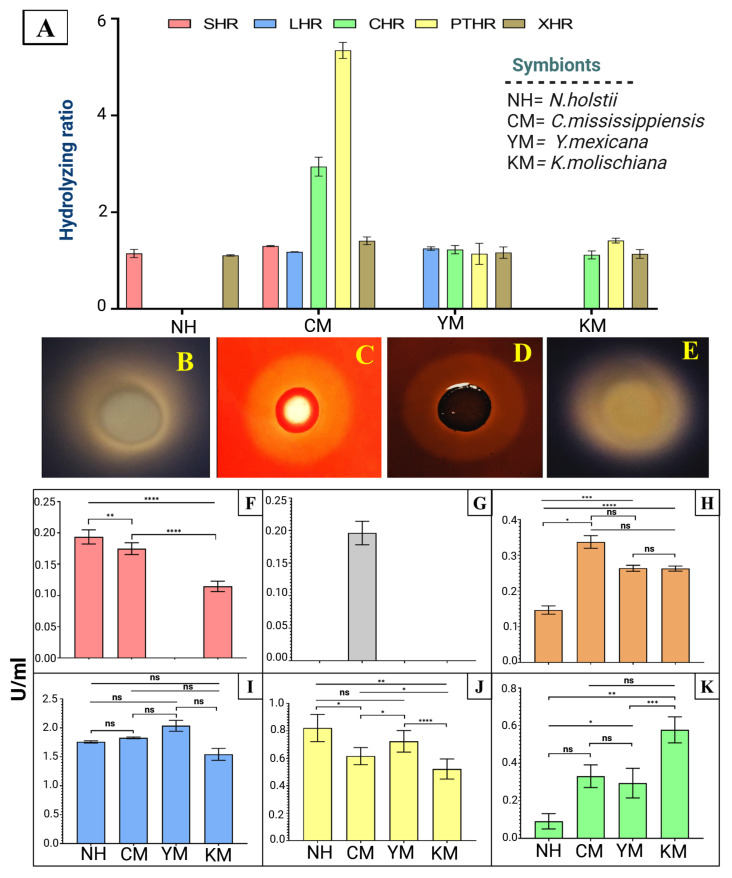
Detection of physiochemical potential of symbiotic yeasts. (**A**) Detection of potential antifungal and digestive enzyme production by symbiotic yeasts. SHR—Starch hydrolysing potential, LHR—Laminarin hydrolysing potential, CHR—Cellulose hydrolysing potential, PTHR—Pectin hydrolysing potential, XHR—Xylan hydrolysing potential. Hydrolysing zone-forming potential of yeast symbionts. (**B**) Starch hydrolysing capability of *Nakazawaea holstii*. (**C**) Starch hydrolysing capability of *Cyberlindnera mississippiensis*. (**D**) Cellulose hydrolysing ability of *C. mississippiensis* (**E**) Pectin hydrolysing efficiency of *C. mississippiensis*. Antifungal and digestive enzyme production capability of symbiotic yeasts (**F**–**K**). (**F**) Amylase production capability of the yeast symbionts. (**G**) β-glucanase production capability of the yeast symbionts. (**H**) Cellulase production capability of the yeast symbionts. (**I**) Chitinase production capability of yeast symbionts. (**J**) Pectinase production capability of the yeast symbionts. (**K**) Xylanase production capability of the yeast symbionts. The standard error of the mean was calculated from three determinations. The significance level of the variables was determined by one-way ANOVA followed by Tukey’s test at a 95% confidence level (*p* < 0.05) using GraphPad Prism software (version 10.4.2). * *p* value < 0.05, ** *p* value < 0.01, *** *p* value < 0.001, **** *p* value < 0.0001, ns—non-significant.

All four yeast strains were assessed for their antifungal activity and digestive enzymatic potential using a qualitative index (QI). The isolated yeast strains exhibited varying degrees of hydrolytic potential against starch, laminarin, cellulose, pectin, and xylan, as indicated by the corresponding qualitative indexes shown in Figure 11A. Notable halo zone formation was observed for starch, cellulose, and pectin hydrolysis, as well as for other tested substrates (Figure 11B–E). No activity was detected for protein or chitin metabolism. Among the yeasts, *N. holstii* demonstrated activities only against starch and xylan. By contrast, *C. mississippiensis* exhibited activities against all substrates. Additionally, the gut isolate *Y. mexicana* displayed activities against laminarin, cellulose, pectin, and xylan, while *K. molischiana* showed activities against cellulose, pectin, and xylan.

#### Antifungal and Digestive Enzyme Production

The four yeast isolates showed varying potential for antifungal and digestive enzyme production (Figure 11F–K). The amylase production capabilities of the gut symbionts *N. holstii*, *C. mississippiensis*, and *K. molischiana* were quantified as 0.193 ± 0.11, 0.175 ± 0.01, and 0.114 ± 0.08 U/mL, respectively (Figure 11F). Notably, β-glucanase production was observed exclusively in *C. mississippiensis* (0.19 ± 0.02 U/mL) (Figure 11G). Similarly, the cellulolytic efficiency of the yeast symbionts was assessed, revealing *C. mississippiensis* as the most prolific cellulase producer (0.33 ± 0.04 U/mL), followed by *K. molischiana* (0.26 ± 0.013 U/mL), *Y. mexicana* (0.25 ± 0.014 U/mL), and *N. holstii* (0.14 ± 0.01 U/mL) (Figure 11H). The yeast isolates *N. holstii*, *C. mississippiensis*, *Y. mexicana*, and *K. molischiana* demonstrated fungal cell wall lytic enzyme (Chitinase) production of 1.77 ± 0.03, 1.82 ± 0.03, 2.04 ± 0.08, and 1.56 ± 0.13 U/mL, respectively (Figure 11I). Furthermore, substantial pectinase production was observed by the four yeast isolates (*N. holstii*—0.84 ± 0.08; *C. mississippiensis*—0.61 ± 0.06; *Y. mexicana*—0.72 ± 0.08, and *K. molischiana* 0.53 ± 0.07 U/mL) (Figure 11J). Additionally, the xylanase production potential of these symbionts was evaluated, with *K. molischiana* emerging as the most efficient producer (0.57 ± 0.07 U/mL), followed by *C. mississippiensis* (0.32 ± 0.06 U/mL), *Y. mexicana* (0.29 ± 0.08 U/mL), and *N. holstii* (0.08 ± 0.04 U/mL) (Figure 11K). However, none of the symbionts produced significant quantities of protease.

## 3. Discussion

The relationship between bark beetles and their associated fungi is intricate and dynamic, encompassing a range of interactions that can vary from mutualism and commensalism to antagonism, depending on the context [37]. These associations may shift from beneficial to harmful, influenced by the life stages of the beetles and changing environmental conditions [38]. To fully understand the context-dependent dynamics in *Ips* bark beetles, it is essential to explore the fungal communities associated with different developmental stages. Moreover, environmental factors and plant–insect interactions play a pivotal role in shaping the microbial communities of insects, including bark beetles [31,39]. This study aimed to investigate the impact of developmental stages and environmental conditions on the fungal community associated with two species of pine-feeding *Ips* bark beetles. We investigated the mycobiome of different developmental stages in pine beetles and their interactions. However, we did not resolve sex-specific microbiota in adult beetles. Sex-dependent variations in microbial associations and their functional significance, therefore, remain open questions.

Our findings revealed the persistence of a core fungal community, including fungal genera such as *Kuraishia*, *Ogataea*, *Ophiostoma*, *Graphilbum*, and *Cyberlindnera*, across the developmental stages of two pine-feeding bark beetles, *Ips acuminatus* and *Ips sexdentatus*. The association of such fungal genera with other *Ips* bark beetles was previously reported [31,39,40]. The occurrence of a persistent fungal community across developmental stages indicates its pivotal role in supporting the host [41,42]. For instance, fungal genera such as *Kuraishia* and *Ogataea* can convert trans and cis-verbenol into anti-aggregation pheromone verbenone, providing a ‘microbe-mediated off switch’ for beetle aggregation, which regulates colony density and reduces intraspecific competition [23]. Additionally, the genome of *K. molischiana* reveals pathways for vitamin B6 and essential amino acids, which may benefit beetle nutrition [43], while *Cyberlindnera* aids in detoxifying pine metabolites and degrading lipids and starch [41]. Similarly, other fungal associates, such as *Ophiostoma*, also have the ability to utilise plant defence compounds as carbon sources [44]. These findings highlight a metabolically adaptable mycobiome that supports host nutrition, detoxification, and chemical signalling, contributing to the beetles’ ecological success.

Developmental stage was associated with clear shifts in fungal richness and diversity. Our study documented greater fungal richness and diversity in gregarious larval stages, suggesting that fungal acquisition aids nutrient assimilation and detoxification [28]. Fungal diversity declined in the pupal stage, likely due to metamorphic changes [45], a pattern also observed in bacterial diversity [46,47]. Notably, *I. sexdentatus* exhibited higher fungal richness and diversity than *I. acuminatus*, reflecting differences in their feeding behaviours. While both species feed on the same pine, *I. acuminatus* targets thin-barked phloem, whereas *I. sexdentatus* feeds on thicker bark. Additionally, *I. acuminatus* clogs maternal tunnels with frass, promoting mutualistic fungus growth in thinner bark areas [48], resulting in distinct microhabitats that likely influence the fungal communities associated with each beetle.

Laboratory adaptation and breeding conditions can strongly influence fungal associations, often producing context-dependent and unpredictable outcomes [49,50]. In the wild, beetles are exposed to diverse environmental pressures that foster richer fungal communities, while controlled lab conditions may induce a bottleneck effect, limiting diversity [50]. Consistent with this, wild-caught *I. acuminatus* exhibited greater fungal richness and diversity compared to lab-bred counterparts, indicating clear differences in mycobiome composition between rearing conditions. However, comparable data for *I. sexdentatus* are currently lacking, warranting further investigation into the ecological drivers behind these differences. Although the present sampling was limited to a single geographic region and included collections from different years, these results provide an initial framework for understanding how wild versus laboratory rearing conditions shape bark beetle mycobiomes and underscore the importance of broader spatial and temporal sampling in future studies.

It is well-documented that insects can acquire microbial communities horizontally as they consume their diet [51,52,53]. Thus, it was expected that the host (wood) microbiome would influence the beetle microbiome. Our findings revealed that beetles shared more amplicon sequence variants (ASVs) with control wood than with gallery wood. This higher sharing with control wood (unfed phloem) suggests microbial acquisition from the wood microbiome during feeding. This phenomenon was particularly notable for *I. acuminatus* beetles, which feed on wood-associated fungi for nutritional benefits [54]. Consequently, differences in the fungal community between fed and unfed wood may reflect changes in the fungal composition driven by beetle feeding. We hypothesise that during wood consumption, bark beetles introduce microbes into the gallery wood microbiome via oral secretions, exoskeleton contact, and faecal deposition, thereby altering the mycobiome. However, further research is required to fully understand the ecological implications of pine microbiome acquisition and exchange in these beetles.

FUNGuild-based functional predictions of the beetle-associated fungal community identified several putative functional groups. The consistent prevalence of saprotrophs across developmental stages highlights their role in facilitating beetle nutrition within a saprophytic environment. These fungi specialise in decomposing decaying organic matter, converting it into usable forms for beetles. Dominant genera, such as *Graphilbum*, *Leptographium*, and *Cryptococcus*, are well-known saprotrophs that derive nourishment from decomposed organic material, including fallen wood [55,56]. Nevertheless, it is crucial to acknowledge that FUNGuild is a software that predicts functional abundances based on marker genes. Therefore, the putative functional prediction of the microbiome requires additional validation through metatranscriptomic, metaproteomic, or other functional assays.

The contrasting growth responses documented here underscore the importance of evaluating bark beetle-associated microbe growth in response to exposure to multiple terpene compounds. While studies tend to challenge microbial resistance against a single terpene, in the field, these microorganisms face a dynamic blend of host monoterpenes that are emitted collectively upon resin flow following beetle attack [57,58]. Our results showed that the monoterpene blend of α-pinene, 3-carene, and terpinolene consistently inhibited growth more than any of the individual compounds. However, it is important to note that this bioassay measured fungal growth under chemical stress and therefore demonstrates differential monoterpene tolerance or sensitivity rather than a detoxification mechanism. The enhanced inhibitory effect of the monoterpene mixture on yeast growth provides a valuable basis for developing testable hypotheses about potential synergistic interactions among terpenoid compounds, which should be examined in future experiments. The early inhibition of *Y. mexicana* and *N. holstii*, followed by partial recovery, indicates that these species may adapt over time but face a competitive disadvantage in high-terpene environments shortly after host colonisation. Future studies combining growth bioassays with targeted chemical analyses, such as GC-MS, will be necessary to quantify terpene depletion in the medium and identify possible degradation or transformation metabolites. Integrating such chemical profiling with metabolomics and transcriptomic analyses would provide deeper insight into whether observed growth responses reflect terpene tolerance, transformation, detoxification, or other physiological stress responses. Overall, testing exposure to terpene mixtures is important because it more accurately reflects the selective pressures that may shape bark beetle-associated microbial communities.

Microscopic imaging of gut colonisation, together with biofilm assays on culturable isolates, indicated that microbial assemblages were more prevalent in the midgut than in the hindgut. This pattern aligns with prior observations in the guts of *Dichelops melacanthus* and *Cephalotes rohweri* [59,60]. Although biofilm formation by gut symbionts has been documented across diverse animals, including fish, reptiles, insects, and humans [61,62,63,64], biofilm-forming yeasts as bark beetle gut symbionts, particularly in *I. sexdentatus*, have yet to be reported. Furthermore, microscopic (SEM) visualisation of the dynamic interactions between gut yeast isolates and entomopathogenic fungi (EPF) or beetle-associated non-pathogenic ophiostomatoid fungi revealed distinct degradation and deformation of EPF mycelia. By contrast, no such structural disorientation was observed in ophiostomatoid fungi. These observations suggest a pattern of selective antagonism in vitro toward entomopathogenic fungi, while allowing coexistence with wood-associated fungi in beetles. However, it is important to note that the antagonism assays were conducted under laboratory plate conditions and therefore do not fully mimic the physicochemical environment of pine galleries, including humidity, pH, resin chemistry, nutrient availability, and spatial structure. Future in vivo studies in which beetles are challenged with entomopathogenic fungi in the presence and absence of candidate symbiotic yeasts, followed by measurements of beetle survival, pathogen colonisation, and yeast persistence, will be necessary to determine whether the observed in vitro antagonism translates into protective effects under ecologically relevant conditions.

Interestingly, the yeast isolate *C. mississippiensis*, which exhibited chitinase and β-glucanase activities, showed extensive disruption of fungal cell walls and mycelial collapse [65]. Moreover, the digestive CAZyme activities of the yeast isolates, such as amylase, cellulase, pectinase, and xylanase, suggest a contributory role in mycelial distortion and cell wall degradation. The antifungal and digestive enzyme activities of the studied yeasts isolated from various sources have been previously reported [43,66,67,68,69]. Hence, the structural deformation of fungal mycelia could plausibly be explained by the ability of the gut isolates to secrete extracellular antifungal and digestive enzymes, although the precise molecular mechanisms remain to be resolved. Nonetheless, biofilm formation in the midgut by the isolates may further potentiate both antifungal and digestive functions. Additionally, the non-entomopathogenic ophiostomatoid fungi that co-occur with bark beetles may be intrinsically less vulnerable under gut-like conditions or effectively buffered by ecological compatibility (e.g., tolerance/neutrality within beetle–fungus consortia). This interpretation is consistent with reports that beetle-associated yeasts preferentially inhibit entomopathogenic fungi [70]. Therefore, the enzyme repertoire associated with gut yeasts appears to exhibit considerable selectivity against entomopathogenic fungi, disrupting EPF mycelia while having little or no detectable effect on the non-entomopathogenic fungi associated with our samples. This pattern suggests a potential form of selective antagonism, whereby gut-associated yeasts may restrict pathogenic fungi while allowing putatively beneficial or commensal fungi to persist. However, targeted in vivo experiments and molecular analyses are needed to confirm this interaction and clarify its mechanistic basis and ecological significance.

The present study advances our understanding of the fungal communities associated with the two pine-feeding bark beetles by integrating developmental, environmental, host-associated, and in vitro interaction perspectives. Nevertheless, a few limitations of the study should be acknowledged. The sample size is relatively limited, with few beetles per replicate, which likely reduced our ability to distinguish persistent residents from transient taxa when delineating the core microbiome. Sampling from a single forest in the Czech Republic further restricts geographic inference and introduces potential environmental confounders that may influence pine beetle-fungal assemblages. Additionally, fungal ITS2 amplicon sequencing constrains taxonomic resolution largely to the genus level, potentially obscuring species- or strain-specific associations with functional relevance. qPCR validation at developmental stages was performed only for *I. sexdentatus*, using a few selected taxa-specific primers, whereas for *I. acuminatus* it was restricted to adult beetles due to limited sample availability.

Despite these limitations, this study provides a foundation for future work and generates testable hypotheses on the ecological roles of fungal associates in bark beetle systems. Larger and geographically broader sampling, combined with higher-resolution approaches such as shotgun metagenomics or strain-resolved sequencing, co-occurrence modelling, targeted metabolomics, GC-MS-based chemical profiling, and functional genetic analyses of fungal isolates, will be important for clarifying the mechanisms underlying bark beetle–fungus–host tree interactions.

## 4. Materials and Methods

### 4.1. Bark Beetle Rearing and Collection

Multiple logs (dbh ~40 cm) infested with two adult *Ips* pine beetles (*Ips sexdentatus* and *Ips acuminatus*) (Coleoptera: Curculionidae: Scolytinae) were collected from different pine trees in the Rouchovany forest area (49.0704° N, 16.1076° E) in the Czech Republic, 2020. Published literature was utilised for the taxonomic identification of both adult beetles. The infested logs collected from the forest were immediately brought to the debarking room, and the adult beetles were collected using surface-sterilised tweezers. To generate laboratory-bred beetles, the field-collected wild adult beetles were then transferred to fresh uninfested logs collected from the same forest area and reared under laboratory conditions (day temperature, 25 °C; night temperature, 19 °C; relative humidity, 60%) until the F2 generation, as described in our previous study [47]. Thus, the F2 beetle samples and their associated gallery wood originated from laboratory-bred beetles derived from wild adults collected in 2020 but developed under controlled laboratory conditions rather than directly in the forest. Approximately 50 samples, representing each life stage (larvae, pupae, and adults) from several infested logs (at least 10 cm below the edges to minimise contamination) were collected in 50 mL conical tubes. These conical tubes were snap-frozen with liquid nitrogen and kept at −80 °C for future metagenomic sample preparation. The gallery wood (Fed.W) was collected from the same F2 generation beetle-infested logs by excising small sections of gallery-associated phloem/wood, comprising both mother and larval galleries, following a standard protocol [71]. Samples were not restricted to superficial surface scrapings. Similarly, uninfested, fresh wood samples (unfed phloem tissue) were collected as a control. Both wood samples were collected using surface-sterilised blades, which were immediately placed in RNAlater and stored at −80 °C until DNA extraction. The size of the individual wood sample replicate was 0.5 by 1 cm sections. In 2021, an additional set of naturally infested logs was collected from different pine trees in the same forest area. These samples represented the wild-collected comparison group. Adult beetles and their corresponding gallery wood samples were collected directly from the same field-infested logs using the same procedures described above. Therefore, the wild beetles and wild gallery wood samples were paired at the log level, whereas the laboratory F2 beetles and F2 gallery wood samples were paired within the same laboratory-reared infested logs. Because wild samples were collected in 2021 and the laboratory F2 colonies were established from wild beetles collected in 2020, temporal effects cannot be completely separated from rearing-condition effects. However, all samples originated from the same forest area, and beetle and wood samples were paired with their respective infested logs wherever possible. Therefore, wild versus laboratory comparisons should be interpreted cautiously as reflecting rearing-related differences potentially influenced by year-associated environmental variation.

### 4.2. DNA Extraction

All bark beetle samples (larvae, pupae, and adults) were randomly selected, and disinfection of the beetles was carried out using 70% ethanol for 1 min, followed by washing in sterile water. This process was repeated three times to remove any surface contaminants. Five biological replicates from each developmental stage and four from each wood sample were selected for fungal ITS2 amplicon sequencing at Novogene, Beijing, China. Because of variations in size, the individuals in each biological replicate differed for both species. For *I. sexdentatus*, one individual per replicate was used. However, for *I. acuminatus*, larvae (4 larvae/replicate), pupae (2 pupae/replicate), and adults (5 adults/replicate) were used. The DNA extraction and quantification procedures were previously reported (Khara et al., 2024) [47]. Briefly, the samples were subjected to total DNA extraction using the NucleoSpin Soil DNA kit (MACHEREY-NAGEL GmbH & Co. KG, Düren, Germany). DNA extraction from wood samples (100–120 mg per replicate) was performed using the DNeasy Plant Mini Kit (Qiagen, Hilden, Germany). The extracted DNA samples underwent purification to eliminate any potential PCR inhibitor using the DNeasy Powerclean Pro cleanup kit (Qiagen, Hilden, Germany). The purified DNA samples were quantitatively and qualitatively assessed using 1% agarose gel electrophoresis and a Qubit 2.0 Fluorometer (Thermo Scientific, Waltham, MA, USA). It is worth noting that for each biological replicate, beetle individuals were pooled prior to DNA extraction, and DNA was extracted from the pooled replicate. Although the number of individuals included per replicate differed between beetle species, the purified DNA was normalised across all samples, and 1 ng/µL DNA was used as a template for each PCR reaction.

### 4.3. Amplicon Sequencing

Diluted purified DNA (1 ng/µL), fungal ITS2 region primers (ITS3, ITS4) [72] tagged with unique barcodes, and Phusion High-Fidelity PCR Master Mix (New England Biolabs, Ipswich, MA, USA) were used to initiate the PCR reactions. Subsequently, a no-template PCR reaction was performed as a negative control to verify the absence of contamination. The PCR amplicons were visualised on a 2% agarose gel, and the Qiagen Gel Extraction Kit (Germany) was used to purify the amplified products. Sequencing libraries were constructed with equidensities of amplified products using the NEBNext Ultra II DNA Library Pre-Kit (Illumina). A Qubit 2.0 Fluorometer (Thermo Fisher Scientific) and Agilent Bioanalyser 2100 system (Santa Clara, CA, USA) were used to check the quality and quantity of the developed libraries. Finally, 250 bp paired-end reads were generated from the libraries using the Illumina NovaSeq 6000 sequencing platform (Illumina, Inc., San Diego, CA, USA).

### 4.4. Bioinformatic Data Analysis

#### 4.4.1. Data Processing and Species Annotation

Bioinformatic analysis of the mycobiome was executed using QIIME2 (version 2022.2) [73]. After the removal of the primer and barcode sequences, Illumina paired-end raw reads were generated using FLASH (V1.2.11) [74]. The quality of the assembled raw reads was checked using ‘fastp’ software (version 0.23.0) to assemble high-quality clean reads. Chimeric sequence detection and removal were executed using VSEARCH software (version 2.7.1) [75] to facilitate downstream bioinformatic analyses. The DADA2 module in QIIME2 [76] was used to remove sequences with fewer than five reads to generate ASVs [77] and the ASV abundance table. The UNITE database (version 9.0) [78] and Classify-sklearn module (version 2020.6) [79] of QIIME2 (version 2022.2) [73] were used to annotate taxa to ASVs. It is important to note here that pooling different numbers of beetles during DNA extraction may influence the probability of detecting rare or low-prevalence fungal ASVs, particularly if such taxa occur only in a subset of individuals. Hence, direct interspecific comparisons should be interpreted with caution, and the focus should be on broad patterns in mycobiome composition rather than on individual-level fungal variations.

#### 4.4.2. Alpha Diversity

Alpha diversity indices were used to assess the richness and diversity of the fungal community within a sample. Indices like community richness (Chao1), evenness (Pielou) [80], diversity (Shannon and Simpson) [80], and Good’s coverage (sequence depth) [81] were estimated using QIIME2 (version 2022.2) and R software (version 3.5.3) [82]. Statistical analysis between each group was performed using the Kruskal–Wallis pairwise group test. To identify the core fungal communities associated with each beetle species, we defined the core mycobiome as the set of amplicon sequence variants (ASVs) detected in at least 60% of samples within a given group. This threshold was chosen to capture consistently occurring taxa while minimising the inclusion of rare or sporadic members. Core ASVs were identified separately for *I. sexdentatus* and *I. acuminatus* across developmental stages and rearing conditions. To evaluate the degree of overlap and specificity in the core mycobiome between the two beetle species, shared and unique core ASVs were then compared using presence–absence analysis.

#### 4.4.3. Beta Diversity

The variation in fungal diversity between different samples was estimated using the UniFrac distance metric [83] with QIIME2 (version 2022.2). Non-metric multidimensional scaling (NMDS) was performed in R software based on measured UniFrac distances [84]. The determination of significant differences in the mycobiome was performed using ADONIS and ANOSIM functions [85,86] in QIIME2 (version 2022.2). ADONIS analysis is a non-parametric multivariate variance test used to reveal significant differences among sample groups [87]. Subsequently, ANOSIM function analysis determines whether the variation between different groups is greater than the within-sample group variation [88]. Differentially abundant fungal ASVs were identified using Metastats (version 2.0) (employing multiple hypothesis tests for sparsely-sampled features and the false discovery rate, FDR) [89] as the primary screening approach, as this method is suitable for microbial community data and evaluates differences in relative abundance while considering variation among biological replicates. Because different microbiome differential-abundance methods can produce variable results, complementary targeted comparisons can help support cautious interpretation of group-wise differences [90]. Accordingly, complementary *t*-tests (*p*-value < 0.05) [91] were applied only as secondary comparisons of mean relative abundance for selected taxa or functional categories and were not used as a replacement for Metastats. Fungal ASVs showing consistent significance across these complementary analyses were interpreted as differentially abundant taxa. Statistically significant biomarkers were identified between the tested samples using LEfSe (linear discriminant analysis effect size) analysis with a predefined threshold of linear discriminant analysis score (LDA score [log10] > 4) [92]. The probable functional/ecologically categorised profile was obtained using FUNGuild software (version 1.0) [93].

### 4.5. Quantitative PCR Assay

The relative abundance of selected fungal taxa was measured using a quantitative PCR (qPCR) assay, following the optimised protocol [47]. For *I. sexdentatus* samples, six individuals were used per replicate for each developmental stage (larvae, pupae, and adults). For *I. acuminatus*, only adults were analysed due to limited sample availability. These qPCR samples corresponded to the same laboratory F2 and wild adult collections described in Section 4.1 and therefore shared the same temporal and contextual structures as the amplicon sequencing dataset. Four biological replicates were prepared for each group. For the qPCR assay, one universal fungal ITS2 primer and four fungal genus-specific primers were used (Table S4). Fungal genus-specific primers for *Nakazawaea*, *Kuraishia*, *Ophiostoma*, and *Ogatea* were designed in-house, using available ITS gene sequences from NCBI. The primer specificity was determined by sequencing the amplified products and confirming the identity through BLAST analysis (http://blast.ncbi.nlm.nih.gov, accessed on 6 November 2025). qPCR reactions were conducted in 10 μL volumes, which included 4 μL of template DNA (10 ng μL^−1^), 5 μL of SYBR^®^ Green PCR Master Mix (Applied Biosystems, Thermo Fisher Scientific, Foster City, CA, USA), and 0.5 μL of each forward and reverse primer (10 μM). Amplification was performed on a QuantStudio thermocycler (Thermo Fisher Scientific, Waltham, MA, USA) with the following conditions: an initial denaturation at 95 °C for 5 min, followed by 40 cycles of denaturation at 95 °C for 15 s and annealing/extension at 60 °C for 30 s. For normalisation, we used housekeeping genes that showed stable expression across samples: β-tubulin for *I. sexdentatus* samples [94] and elongation factor 1-α (EF1α) and ribosomal protein (RPL7) for *I. acuminatus* samples (unpublished data). We calculated relative quantification (RQ) using the 2^−ΔΔCt^ method, where ΔCt indicates the difference between the Ct value of the target taxon and the reference gene. This method estimates fold changes in target abundance compared to the reference gene, avoiding inconsistencies that can occur when using the total fungal population as a denominator [95]. We conducted statistical analysis of relative abundance in R (version 4.3.1) using ANOVA, followed by post hoc tests and *t*-tests to identify significant differences between groups [82]. Furthermore, we correlated the qPCR results with the abundance data from metagenomic sequencing.

### 4.6. Fungal Cultures and Identification

Fungal species were isolated from healthy pine beetles as well as pine wood samples. The beetles were disinfected using 70% ethanol for 1 min, followed by three washes in sterile water to remove any surface contaminants. The beetles were then crushed in PBS solution, and serial dilutions (10^−1^ to 10^−6^) were prepared. The dilutions were spread on potato dextrose agar (PDA) plates and incubated at 28 °C for 5 days. The wood samples were cut into small sections of 0.5 cm and placed on the PDA plates. The fungal growth observed was subcultured three times to obtain pure culture isolates. Similarly, the pathogenic fungal mycelia from the surface of the dead beetles were extracted and cultured on PDA plates. The fungal isolates were identified using Sanger sequencing and NCBI BLAST analysis. The sequences were submitted to the NCBI GenBank database with accession numbers PX491830–PX491839 (Table S5). Although the identity of selected cultured yeast isolates was confirmed using ITS-Sanger sequencing, multilocus phylogenetic analysis was not performed. Therefore, species-level assignments should be interpreted cautiously where ITS resolution is limited.

### 4.7. Monoterpene BIOASSAY

Four yeast strains (*Yamadazyma mexicana*, *Nakazawaea holstii*, *Kuraishia molischiana*, and *Cyberlindnera mississippiensis*) isolated from the two pine-feeding beetles were used in the bioassay with monoterpenes to understand their impact on yeasts. We assayed three selected monoterpenes (α-pinene, 3-carene, and terpinolene) and a monoterpene blend (a mixture of α-pinene, 3-carene, and terpinolene) at varying concentrations to mimic the chemical exposure conditions in nature within the host environment. Monoterpene concentrations were considered and calculated in accordance with earlier publications [34]. For α-pinene, we used concentrations of 100 ng µL^−1^, 500 ng µL^−1^, and 1500 ng µL^−1^; for 3-carene, 100 ng µL^−1^, 500 ng µL^−1^, and 1000 ng µL^−1^; and for terpinolene, 50 ng µL^−1^, 100 ng µL^−1^, and 200 ng µL^−1^. The monoterpene blend consisted of 1500 ng µL^−1^ α-pinene, 1000 ng µL^−1^ 3-carene, and 100 ng µL^−1^ terpinolene. Dimethyl sulfoxide (DMSO, 0.5% (*v*/*v*)) was utilised as the control solvent since a concentration of that amount was required to assist in dissolving the monoterpenes in potato dextrose broth (PDB) medium. Overnight yeast cultures adjusted to an optical density (OD_600_) of 0.5 were used to inoculate the growth assay. Spectrophotometric measurements at OD_600_ were taken at fixed intervals of time. The experiments were carried out in triplicate to facilitate reproducibility and consistency of data.

### 4.8. Scanning Electron Microscopy (SEM)

#### 4.8.1. Gut Colonisation of Fungi

To explore *I. sexdentatus* gut microbial colonisation, the dissected midgut and hindgut were fixed in 2.5% glutaraldehyde in 0.2 M cacodylate buffer (pH 7.0) at 4 °C for 7 days. The gut samples were then dehydrated using varying concentrations of ethanol wash (35%, 50%, 70% and 96%), with an incubation period of 10 min in each step. The samples were fixed to a cylinder with electron-conductive double-sided adhesive carbon tabs (EM-Tec CT6, Micro to Nano BV, Haarlem, The Netherlands.) and coated with gold (10 nm thickness) using a JFC 1300 Auto Fine Coater (JEOL). The samples were then observed under a JSM-IT500HR InTouchScope™ scanning electron microscope (JEOL, Akishima, Tokyo, Japan) at an accelerating voltage of 3 kV with a working distance of 11–13.1 mm.

#### 4.8.2. Biofilm Formation

The biofilm-forming capability of the gut isolates was also examined under SEM (JEOL). The four yeast isolates were grown in potato dextrose broth (PDB) and also in starch-, chitin-, gelatin-, cellulose-, laminarin-, pectin-, and xylan-supplemented broth (Table S6) under static conditions for 24–48 h at 28 °C, with small pieces of glass chips (1 × 1 cm) placed at the bottom of the conical flask. After incubation, the yeast film formed on the glass chips at the lower portion of the media was collected and gently washed twice with sterile PBS (1×), followed by dehydration with an ethanol gradient (5–100% at 5% increments for 1 min each). The samples were fixed to a cylinder, coated, and examined under SEM using the same parameters as previously mentioned.

#### 4.8.3. Antifungal Activity

To decipher the antifungal activity of the yeast isolates against the entomopathogenic fungal (EPF) strains and non-entomopathogenic fungal (NEPF) strains (Table S5), the mycelial morphology was visualised under SEM. Fungal mycelia of pathogenic and non-pathogenic strains, as well as yeast symbionts, were obtained from pure cultures grown on PDB for 7 days and overnight (optical density (OD) of 0.5 at 600 nm), respectively. Alternatively, co-cultures of EPF–yeast and NEPF–yeast were prepared by inoculating EPF or NEPF grown on PDB for 7 days at 28 °C with an overnight-grown yeast culture (optical density (OD) of 0.5 at 600 nm) in PDB and incubating for 48 h at 28 °C. The experimental setup included one EPF or NEPF that was co-cultured with one yeast at a time. The samples were fixed overnight in 2.5% glutaraldehyde in 0.2 M cacodylate buffer (pH 7.0) at 4 °C. Subsequently, the fixed mycelia were dehydrated using a series of ethanol washes with increasing concentrations of ethanol (10–95%), with a five-minute incubation for each step. The experiment used 3 biological replicates, each with 3 technical replicates, resulting in a total of 9 replicates.

### 4.9. Enzyme Production Assay

The ability of the yeast isolates to produce antifungal enzymes (chitinase, protease, and β-glucanase) and digestive enzymes (amylase, cellulase, pectinase, and xylanase) was evaluated spectrophotometrically based on standard protocols [96,97,98,99,100,101,102]. Four yeast isolates were grown on specific agar media (Table S6) and incubated at 28 °C for 48 h. The antifungal and digestive enzyme-producing capabilities of the gut isolates were assessed using a qualitative index (QI). The experiment used 3 biological replicates, each with 3 technical replicates, resulting in a total of 9 replicates.

#### 4.9.1. Estimation of Antifungal Enzymes

The activities of selected antifungal enzymes (chitinase, protease, and β-glucanase) produced by the yeast isolates were quantified (U mL^−1^) spectrophotometrically using standard substrate assays. Chitinase activity was assayed with 1% (*w*/*v*) colloidal chitin in phosphate buffer (pH 7.0) as the substrate; one unit (U) was defined as the amount of enzyme releasing 1 mM N-acetylglucosamine (GlcNAc) per hour at 28 °C [99]. Protease activity was determined using 1% (*w*/*v*) casein in phosphate buffer (pH 7.0), following a published protocol [103], where one unit was defined as the amount of enzyme liberating 1 µM tyrosine equivalents from casein at 28 °C. While β-glucanase activity was measured according to a published protocol [104], using 1% (*w*/*v*) laminarin in phosphate buffer (pH 7.0) as the substrate, where one unit was defined as the amount of enzyme releasing 1 mM glucose per minute at 28 °C. For each assay, the corresponding reaction mixture prepared with uninoculated production medium served as the control. The experiment used 3 biological replicates, each with 3 technical replicates, resulting in a total of 9 replicates.

#### 4.9.2. Estimation of Digestive Enzymes

The activities of selected digestive enzymes (amylase, cellulase, pectinase, and xylanase) were quantified (U mL^−1^) spectrophotometrically using the dinitrosalicylic acid (DNS) assay. Amylase activity was assayed according to ref. [105] using 1% (*w*/*v*) soluble starch in phosphate buffer (pH 7.0) as the substrate; one unit (U) was defined as the amount of enzyme releasing 1 mM glucose per minute at 28 °C. Cellulase activity was measured using 1% (*w*/*v*) carboxymethyl cellulose (CMC) in phosphate buffer (pH 7.0); reducing sugars were quantified at 540 nm using glucose as the standard and reported as U mL^−1^, where one unit corresponded to 1 mM glucose released per minute at 28 °C [105]. Similarly, pectinase activity was determined using 1% (*w*/*v*) citrus pectin in phosphate buffer (pH 7.0) as the substrate; one unit was the amount of enzyme releasing 1 mM glucose per minute at 28 °C [106]. Xylanase activity was measured according to ref. [107] using 1% (*w*/*v*) birchwood xylan in phosphate buffer (pH 7.0); one unit was defined as the amount of enzyme releasing 1 mM glucose per minute at 28 °C. For all assays, reaction mixtures prepared with uninoculated production medium served as controls. The experiment used 3 biological replicates, each with 3 technical replicates, resulting in a total of 9 replicates.

## 5. Conclusions

This study provides new insights into the fungal communities associated with *I. sexdentatus* and *I. acuminatus* across developmental stages, rearing conditions, and gallery substrates. Amplicon-based profiling revealed clear variations in mycobiome composition and diversity across beetle development, rearing environment, and beetle–tree interactions. Larvae generally harboured higher fungal diversity than pupae and adults, while wild-collected beetles showed richer fungal communities than laboratory-bred individuals. The overlap between beetle- and wood-associated mycobiomes suggests microbial exchange during feeding and gallery formation, with saprotrophic fungi representing a dominant functional group. Furthermore, the putative functional predictions and in vitro assay results suggest that some fungal associates may be involved in processes related to nutrient acquisition, detoxification, chemical interactions, or antagonism against entomopathogenic fungi. However, these potential roles should be interpreted cautiously, as they require direct in vivo functional validation. Monoterpene blends produced stronger inhibitory effects on yeast symbionts than individual compounds, providing a useful basis for future studies testing potential additive or synergistic interactions using appropriate dose–response and synergy models. Similarly, selective effects against entomopathogenic fungi were observed in vitro, and the enzyme activity assay results together with the SEM observations provide preliminary support for the hypothesis that gut-associated yeasts may contribute to antagonistic activity against fungal pathogens. Nevertheless, the mechanisms underlying these interactions demand dedicated studies.

Overall, the findings provide a framework for generating testable hypotheses about the ecological roles of bark beetle-associated fungi.

## Figures and Tables

**Table 1:** Sample descriptions.

Sample Code	Sample Details	Collection
Ctrl.W	Uninfested pine wood control for lab rearing	Under lab environment
Fed.W	*Ips sexdentatus*-infested gallery wood from F2 generation	Under lab environment
ISX.Larvae	*Ips sexdentatus* larvae from F2 generation	Lab-bred
ISX.Pupae	*Ips sexdentatus* pupae from F2 generation	Lab- bred
ISX.Adult	*Ips sexdentatus* adults from F2 generation	Lab-bred
ISX.WL.Adult	*Ips sexdentatus* wild-collected adults	Collected from forest
ISX.Ctrl.W	Uninfested wood control for *Ips sexdentatus*	Collected from forest
ISX.Fed.W	*Ips sexdentatus*-infested gallery wood	Collected from forest
IAC.Larvae	*Ips acuminatus* larvae from F2 generation	Lab-bred
IAC.Pupae	*Ips acuminatus* pupae from F2 generation	Lab-bred
IAC.Adult	*Ips acuminatus* adult from F2 generation	Lab-bred
IAC.WL.Adult	*Ips acuminatus* wild-collected adults	Collected from forest
IAC.Ctrl.W	Uninfested wood control for *Ips acuminatus*	Collected from forest
IAC.Fed.W	*Ips acuminatus*-infested gallery or fed wood	Collected from forest

**Table 2:** Metastat analysis revealing the top 10 differentially abundant fungal genera across different developmental stages of *Ips* pine beetles. Taxa with FDR-adjusted *p* < 0.05 were considered significant.

Group		Significantly Different Present Fungal Genera (Top 10 Most Abundant, *p* < 0.05)	
Group 1	Group 2	Significantly Different in Group 1	Significantly Different in Group 2
ISX.Larvae	ISX.Adult	*Clonostachys*, *Paratritirachium*, *Oidiodendron,* *Talaromyces*, *Endoconidiophora*, *Acremonium*	*Ogataea*, *Wickerhamomyces*, *Cryptococcus*, *Candida*
ISX.Larvae	ISX.Pupae	*Penicillium*, *Paratritirachium*, *Oidiodendron,* *Talaromyces*, *Acremonium*, *Endoconidiophora,* *Scoliciosporum*, *Lecanora*	*Ogataea*, *Cryptococcus*
ISX.Pupae	ISX.Adult	*Crumenulopsis*, *Acremonium*, *Rhexographium*, *Endoconidiophora*, *Neonectria*, *Infundichalara*, *Mollisia*	*Nakazawaea*, *Wickerhamomyces*, *Cryptococcus*
IAC.Larvae	IAC.Adult	*Kuraishia*, *Ophiostoma*, *Cyberlindnera*, *Leptographium*, *Cryptococcus*, *Pseudogymnoascus*	*Graphilbum*, *Atractiella*, *Ogataea*, *Trichoderma*
IAC.Larvae	IAC.Pupae	*Nakazawaea*, *Cryptococcus*, *Kuraishia*, *Penicillium*, *Wickerhamomyces*, *Aspergillus*, *Peterozyma*	*Graphilbum*, *Ceratocystiopsis*
IAC.Pupae	ISX.Adult	*Cyberlindnera*, *Crumenulopsis*, *Pseudogymnoascus*, *Ophiostoma*, *Botrytis*	*Dichotomopilus*
ISX.Adult	IAC.Adult	*Ophiostoma*, *Ogataea*, *Kuraishia*, *Cyberlindnera*, *Wickerhamomyces*, *Cryptococcus*, *Crumenulopsis*, *Candida*	*Graphilbum*, *Graphium*
ISX.Larvae	IAC.Larvae	*Ogataea*, *Clonostachys*, *Paratritirachium*, *Talaromyces*, *Oidiodendron*, *Acremonium*	*Graphilbum*, *Nakazawaea*, *Kuraishia*, *Cryptococcus*
ISX.Pupae	IAC.Pupae	*Ogataea*, *Cyberlindnera*, *Crumenulopsis*, *Cryptococcus*, *Oidiodendron*, *Acremonium*, *Wickerhamomyces*, *Diplodia*, *Rhexographium*	*Graphilbum*
ISX.WL.Adult	ISX.Adult	*Leptographium*, *Peterozyma*, *Myxozyma*, *Cordyceps*	*Ogataea*, *Kuraishia*, *Wickerhamomyces*, *Cryptococcus*, *Clonostachys*, *Talaromyces*
IAC.WL.Adult	IAC.Adult	*Myxozyma*, *Ogataea*, *Kuraishia*, *Therrya*, *Saccharomycopsis*, *Cyberlindnera*, *Ophiostoma*, *Clonostachys*	*Graphilbum*, *Atractiella*

## Data Availability

The original data presented in the study are openly available in NCBI Bio under project PRJNA854812.

## Supplementary Materials

The following supporting information can be downloaded at: https://www.mdpi.com/article/10.3390/ijms27104526/s1 [102,108,109,110,111,112,113,114].
