# Supplementary material for: Amplicon-Based Profiling of Fungal Communities Associated with Scots Pine Bark Beetles: Selective Antagonism and Monoterpene Tolerance
Source: Int J Mol Sci. 2026 May 18;27(10):4526. doi: 10.3390/ijms27104526 (PMC13208007; doi:10.3390/ijms27104526)
Supplement: Supplementary file 1 [file ijms-27-04526-s001.zip › Supplementary Tables.pdf]

Table S1| Alpha diversity indices representing fungal richness, evenness, diversity, and sequencing coverage (Good's coverage). The data represented the mean value ±SE for five biological replicates across different life stages of two pine-feeding beetles and four biological replicates for the associated wood samples. (SE-Standard Error).

Samples	Good's coverage (%)	Observed species	Chao1	Pielou	Shannon	Simpson
Control Wood (Ctrl.W)	100	124.5±25.03	125.01±25.14	0.35±0.03	2.39±0.26	0.58±0.06
Fed Wood (Fed.W)	99.97	95.75 ± 13.93	102.56 ± 18.09	0.42 ± 0.05	2.76 ± 0.37	0.72 ±0.08
Ips acuminatus Larvae (IAC.Larvae)	99.96	143.2±14.32	155.74±15.44	0.45±0.06	3.19±0.14	0.79±0.02
Ips acuminatus Pupae (IAC.Pupae)	99.98	129.6±21.74	139.84±26.21	0.37±0.04	2.55±0.29	0.6±0.06
Ips acuminatus Adult (IAC.Adult)	99.98	108.4±2.98	119.08±3.43	0.35±0.02	2.37±0.17	0.61±0.05
Ips acuminatus Wild-type Adult (IAC.WL.Adult)	99.98	172.2±8.34	180.69±8.81	0.53±0.01	3.94±0.12	0.87±0.01
Ips acuminatus Control Wood (IAC.Ctrl.W)	99.9	312.5±47.79	324.13±45.29	0.46±0.09	3.81±0.82	0.72±0.12
Ips acuminatus Fed Wood (IAC.Fed.W)	99.97	117.25±8.29	124.69±11.07	0.48±0.09	3.26±0.57	0.74±0.11
Ips sexdentatus Larvae (ISX.Larvae)	99.9	288.4±40.92	308.84±45.22	0.51±0.03	4.2 ± 0.38	0.85±0.04
Ips sexdentatus Pupae (ISX.Pupae)	99.98	160±19.48	164.52±20.98	0.51±0.04	3.7±0.33	0.82±0.05
Ips sexdentatus Adult (ISX.Adult)	99.98	148.6±6.14	155.95±8.1	0.48±0.04	3.46±0.27	0.8±0.06
Ips sexdentatus Wild-type Adult (ISX.WL.Adult)	99.98	120.4±8.81	123.32±9.34	0.50±0.03	3.46±0.18	0.83±0.02
Ips sexdentatus Control Wood (ISX.Ctrl.W)	100	157±34.66	160.15±34	0.48±0.02	3.46±0.31	083±0.02
Ips sexdentatus Fed Wood (ISX.Fed.W)	100	185.75±28.64	191.21±27.41	0.51±0.00	3.8±0.13	0.87±0.01

Table S2| ADONIS analysis using unweighted UniFrac distances to comprehend significant differences in the fungal communities across developmental stages of both *Ips* pine beetles (ISX-*I. sexdentatus*, IAC-*I. acuminatus*) and wood samples (Df = degree of freedom, SS = sums of squares of deviations, MS = SS/Df, F. Model = F-test value, R2 = the ratio of grouping variance and total variance).

	Df	SumsOf Sqs	Mean Sqs	F.Model	R2	Pr(>F)
**group1**	13	7.145752	0.549673	6.144648	0.615031	0.001
**Residuals**	50	4.472781	0.089456	NaN	0.384969	NaN
**Total**	63	11.618533	NaN	NaN	1.000000	NaN

Table S3| ANOSIM analysis revealing significant variation in the fungal communities across different developmental stages of both *Ips* pine beetles (ISX-*I. sexdentatus*, IAC-*I. acuminatus*). The R values closer to 1.0 denotes significant differences in fungal communities between different developmental stages of both beetles. P value < 0.05 denotes the statistically significant difference.

Group	R-value	P-value
ISX.Adult vs ISX.Larvae	0.856	0.009
ISX.Adult vs ISX.Pupae	0.412	0.009
ISX.Larvae vs ISX.Pupae	0.316	0.024
IAC.Adult vs IAC.Larvae	0.444	0.019
IAC.Adult vs IAC.Pupae	0.208	0.029
IAC.Adult vs ISX.Adult	1	0.009
IAC.Larvae vs ISX.Larvae	0.924	0.024
IAC.Pupae vs ISX.Pupae	0.832	0.014
ISX.Adult vs ISX.WL.Adult	1	0.004
IAC.Adult vs IAC.WL.Adult	1	0.019
IAC.WL.Adult vs ISX.WL.Adult	1	0.004
ISX.Ctrl.W vs ISX.Fed.W	0.75	0.019
ISX.Ctrl.W vs ISX.WL.Adult	0.6125	0.004
ISX.Fed.W vs ISX.WL.Adult	1	0.009
IAC.Ctrl.W vs IAC.Fed.W	0.927	0.02
IAC.Ctrl.W vs IAC.WL.Adult	0.925	0.01
IAC.Fed.W vs IAC.WL.Adult	0.843	0.014

Table S4|Selected fungal primers used for the real-time quantitative PCR assay.

Primer Name	Sequence (5'-3')	Amplicon length (bp)	Annealing temperature (℃)	Reference
LSU	LR3R: GTCTTGAAACACGGACC<br>LR6: CGCCAGTTCTGCTTACC	500	60	(Raja et al., 2017; Vilgalys, 2018)
Nakazawaea	*Nakazawaea*_Fwd: GTTCTGGTGTTGAGTGATACTC<br>*Nakazawaea*_Rev: TCAATCAACGAGTTGGATAAA	123	60	In house
Kuraishia	*Kuraishia*_Fwd: TCGGGTTTGGTGTTGAGTGA<br>*Kuraishia*_Rev: AACTTGTTAGGCCGGGTCTG	175	60	In house
Ophiostoma	*Ophiostoma*_Fwd: TGGTGTTGGGGCTCCTCT<br>*Ophiostoma*_Rev: GCGCGATTTGAACTGGC	140	60	In house
Ogataea	*Ogataea*_Fwd: GGTTTGGTGATGAGCGATACT<br>*Ogataea*_Rev: GCAGAGCCAAAACCATGTAAT	153	60	In house

Table S5| List of fungal isolates.

Strain number	Genus	Source of isolation	NCBI GenBank accession no.
IACA10-22.2	*Nakazawaea holstii*	*Ips acuminatus*	*PX491830*
IACA10-24.4	*Yamadazyma mexicana*	*Ips acuminatus*	*PX491831*
IACB10-15.3	*Kuraishia molischiana*	*Ips acuminatus*	*PX491832*
IACA2.3	*Cyberlindnera mississippiensis*	*Ips acuminatus*	*PX491833*
EP1	*Clonostachys rosea*	Infected Pine logs with *I. acuminatus*	*PX491834*
EP2	*Beauveria bassiana*	Infected Pine logs with *I.acuminatus*	*PX491835*
EP5	*Absidia caatinguensis*	Infected Pine logs with *I.acuminatus*	*PX491836*
EP9	*Trichoderma sp.*	Infected Pine logs with *I. acuminatus*	*PX491837*
A9	*Ophiostoma hongxingense*	Spruce logs infected *I. typographus*	*PX491838*
B4	*Ophiostoma piceae*	Spruce logs infected *I. typographus*	*PX491839*

Table S6|Composition of media used for different enzyme production

**Amylase producing medium (Gandotra et al., 2018)**	
**Ingredients**	**Quantity (g/L)**
Soluble starch	10.00
$NaNO_3$	2.0
$MgSO_4$, $7H_2O$	0.5
K_2HPO_4	0.05
$FeSO_4$, $7H_2O$	0.01
$CaCl_2$, $2H_2O$	0.02
$MnSO_4$, H_2O	0.002
Final pH (at 25 °C)	7.0 ± 0.2
**β-glucanase producing medium (Wu et al., 2018)**	
**Ingredients**	**Quantity (g/L)**
K_2HPO_4	1.00
$NaNO_3$	3.00
KCl	0.5
$MgSO_4$, $7H_2O$	0.5
$FeSO_4$, $7H_2O$	0.5
Laminarin	10.00
Final pH (at 25 °C)	7.0 ± 0.2
Agar agar	20.00
**Cellulase producing medium (Saha et al., 2006)**	
**Ingredients**	**Quantity (g/L)**
Carboxymethyl Cellulose (CMC)	10
K_2HPO_4	04
Na_2HPO_4	04
Tryptophan	02
$CaCl_2$	0.2
$MgSO_4$, $7H_2O$	0.001
Final pH (at 25 °C)	7.0 ± 0.2
Agar agar	20.00
**Chitinase producing medium (Kuddus & Ahmad, 2013)**	
**Ingredients**	**Quantity (g/L)**
Na_2HPO_4	6.00
KH_2PO_4	3.00
NH_4Cl	1.00
NaCl	0.5
Yeast Extract	0.05
Chitin	10.00

Final pH (at 25 °C)	7.2± 0.2
Agar agar	20.00
**Pectin producing medium (Prem Anand et al., 2010)**	
**Ingredients**	**Quantity (g/L)**
Citrus pectin	10
NaNO3	2.0
MgSO4, 7H2O	0.5
K2HPO4	0.05
FeSO4	0.01
CaCl2	0.02
MnSO4	0.002
Final pH (at 25 °C)	7.0 ± 0.2
Agar agar	20.00
**Protease producing medium (Banerjee & Ray, 2017)**	
**Ingredients**	**Quantity (g/L)**
Peptone	5.0
Gelatin	4.0
Beef Extract	3.0
Final pH (at 25 °C)	7.0 ± 0.2
Agar agar	20.00
**Xylanase producing medium (Anand et al., 2013)**	
**Ingredients**	**Quantity (g/L)**
Xylan	10
$NaNO_3$	2.0
$MgSO_4$, $7H_2O$	0.5
K_2HPO_4	0.05
$FeSO_4$	0.01
$CaCl_2$	0.02
$MnSO_4$	0.002
Final pH (at 25 °C)	7.0 ± 0.2
Agar agar	20.00

References:

Anand, A., Kumar, V., & Satyanarayana, T. (2013). Characteristics of thermostable endoxylanase and β-xylosidase of the extremely thermophilic bacterium Geobacillus thermodenitrificans TSAA1 and its applicability in generating xylooligosaccharides and xylose from agro-residues. *Extremophiles*, *17*(3), 357-366.

Banerjee, G., & Ray, A. K. (2017). Impact of microbial proteases on biotechnological industries. *Biotechnology and genetic engineering reviews*, *33*(2), 119-143.

Gandotra, S., Bhuyan, P. M., Gogoi, D. K., Kumar, A., & Subramanian, S. (2018). Screening of nutritionally important gut bacteria from the lepidopteran insects through qualitative enzyme assays. *Proceedings of the National Academy of Sciences, India Section B: Biological Sciences*, *88*(1), 329-337.

Kuddus, M., & Ahmad, I. (2013). Isolation of novel chitinolytic bacteria and production optimization of extracellular chitinase. *Journal of Genetic Engineering and Biotechnology*, *11*(1), 39-46.

Prem Anand, A. A., Vennison, S. J., Sankar, S. G., Gilwax Prabhu, D. I., Vasan, P. T., Raghuraman, T., Jerome Geoffrey, C., & Vendan, S. E. (2010). Isolation and characterization of bacteria from the gut of Bombyx mori that degrade cellulose, xylan, pectin and starch and their impact on digestion. *Journal of Insect Science*, *10*(1), 107.

Raja, H. A., Miller, A. N., Pearce, C. J., & Oberlies, N. H. (2017). Fungal identification using molecular tools: a primer for the natural products research community. *Journal of natural products*, *80*(3), 756-770.

Saha, S., Roy, R. N., Sen, S. K., & Ray, A. K. (2006). Characterization of cellulase-producing bacteria from the digestive tract of tilapia, Oreochromis mossambica (Peters) and grass carp, Ctenopharyngodon idella (Valenciennes). *Aquaculture Research*, *37*(4), 380-388.

Vilgalys, R. (2018). Conserved primer sequences for PCR amplification of fungal rDNA. In: Duke University, USA.

Wu, Q., Dou, X., Wang, Q., Guan, Z., Cai, Y., & Liao, X. (2018). Isolation of β-1, 3-glucanase-producing microorganisms from Poria cocos cultivation soil via molecular biology. *Molecules*, *23*(7), 1555.
