# Supplementary figures and images for: Amplicon-Based Profiling of Fungal Communities Associated with Scots Pine Bark Beetles: Selective Antagonism and Monoterpene Tolerance
Source: Int J Mol Sci. 2026 May 18;27(10):4526. doi: 10.3390/ijms27104526 (PMC13208007; doi:10.3390/ijms27104526)

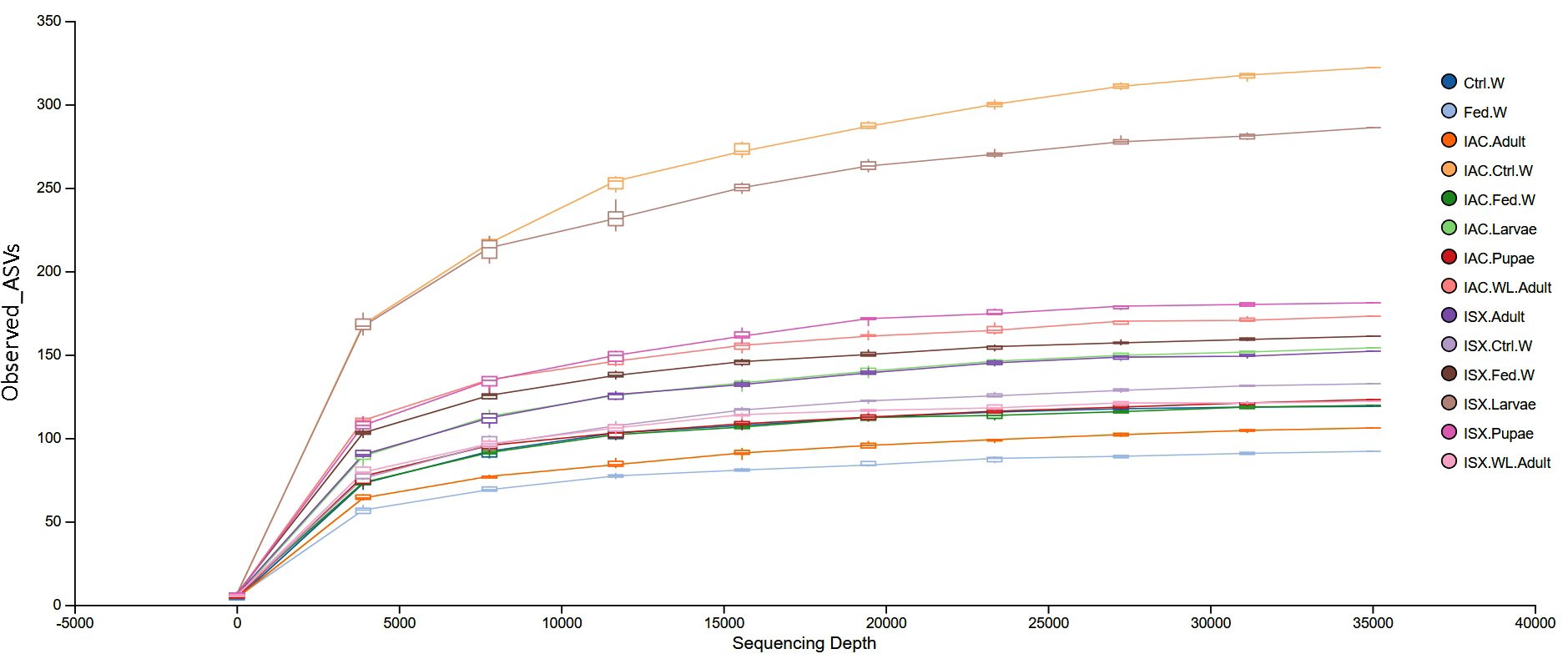

Supplement: Supplementary file 1 [file ijms-27-04526-s001.zip › Figure S1.tif]

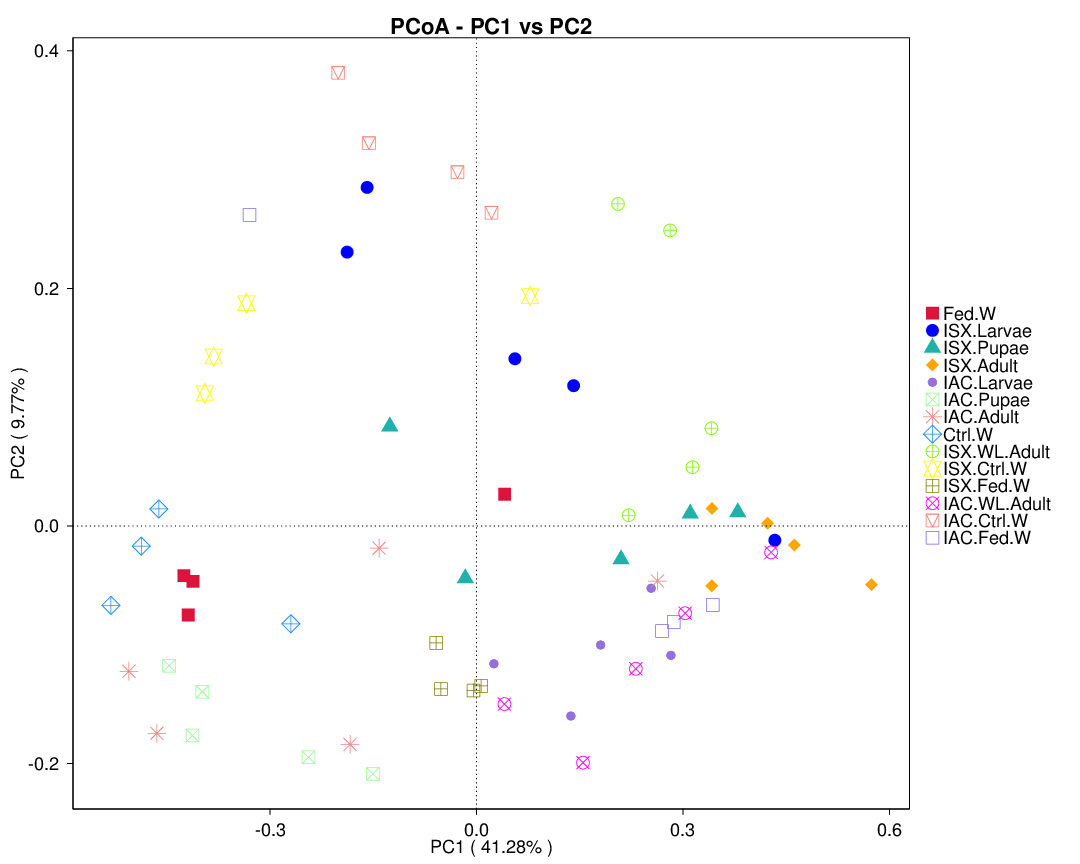

Supplement: Supplementary file 1 [file ijms-27-04526-s001.zip › Figure S2.png]

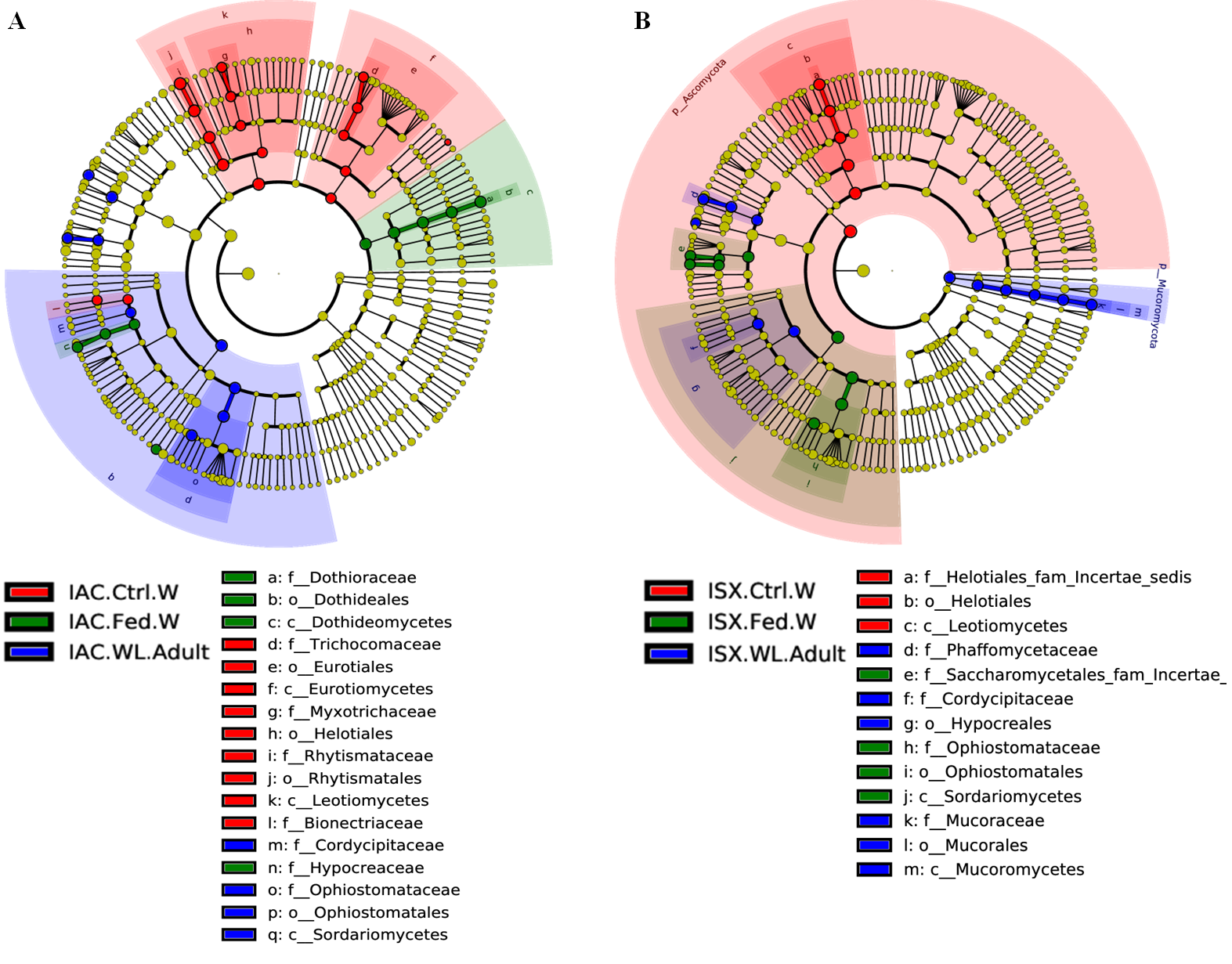

Supplement: Supplementary file 1 [file ijms-27-04526-s001.zip › Figure S3.tif]

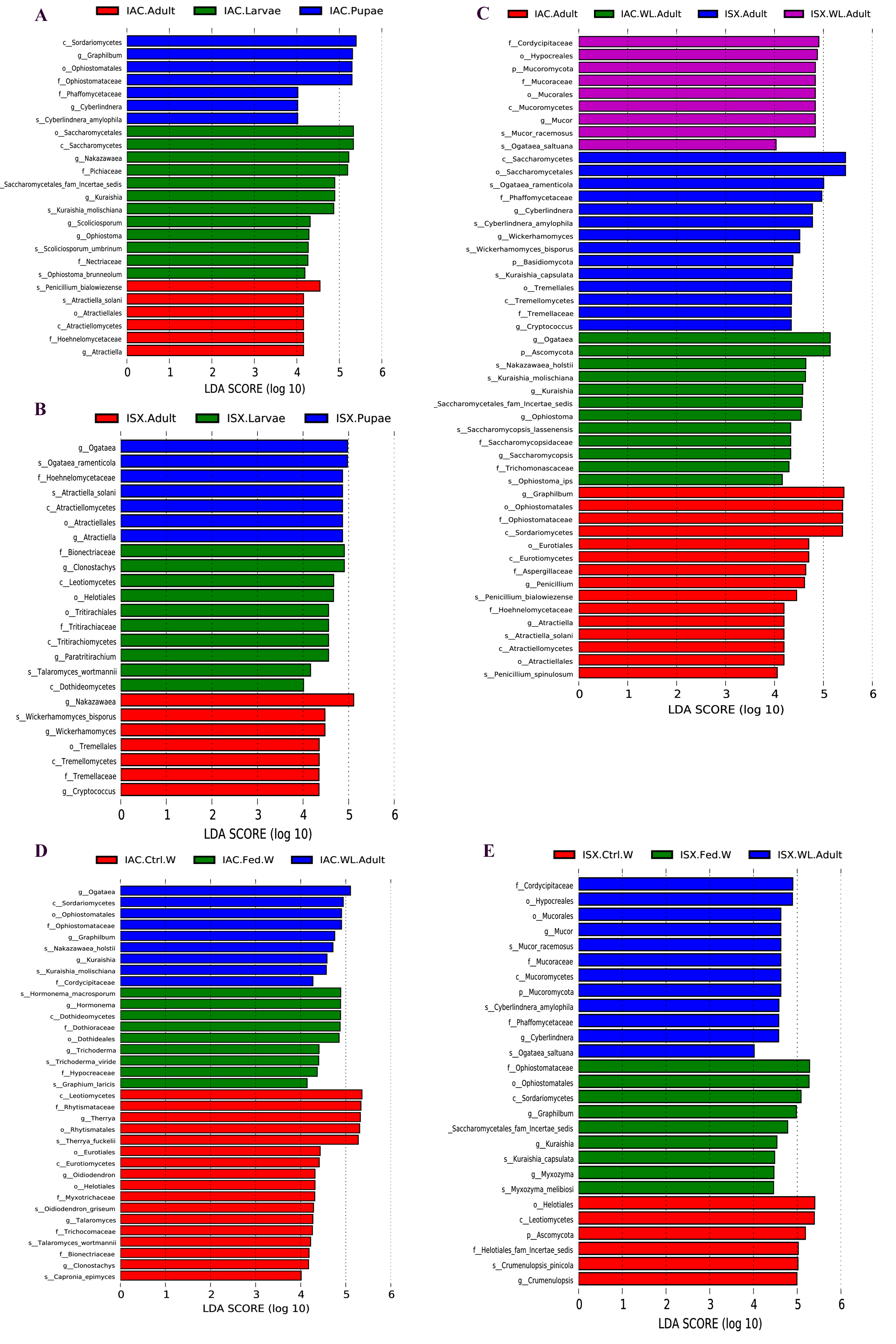

Supplement: Supplementary file 1 [file ijms-27-04526-s001.zip › Figure S4.tif]

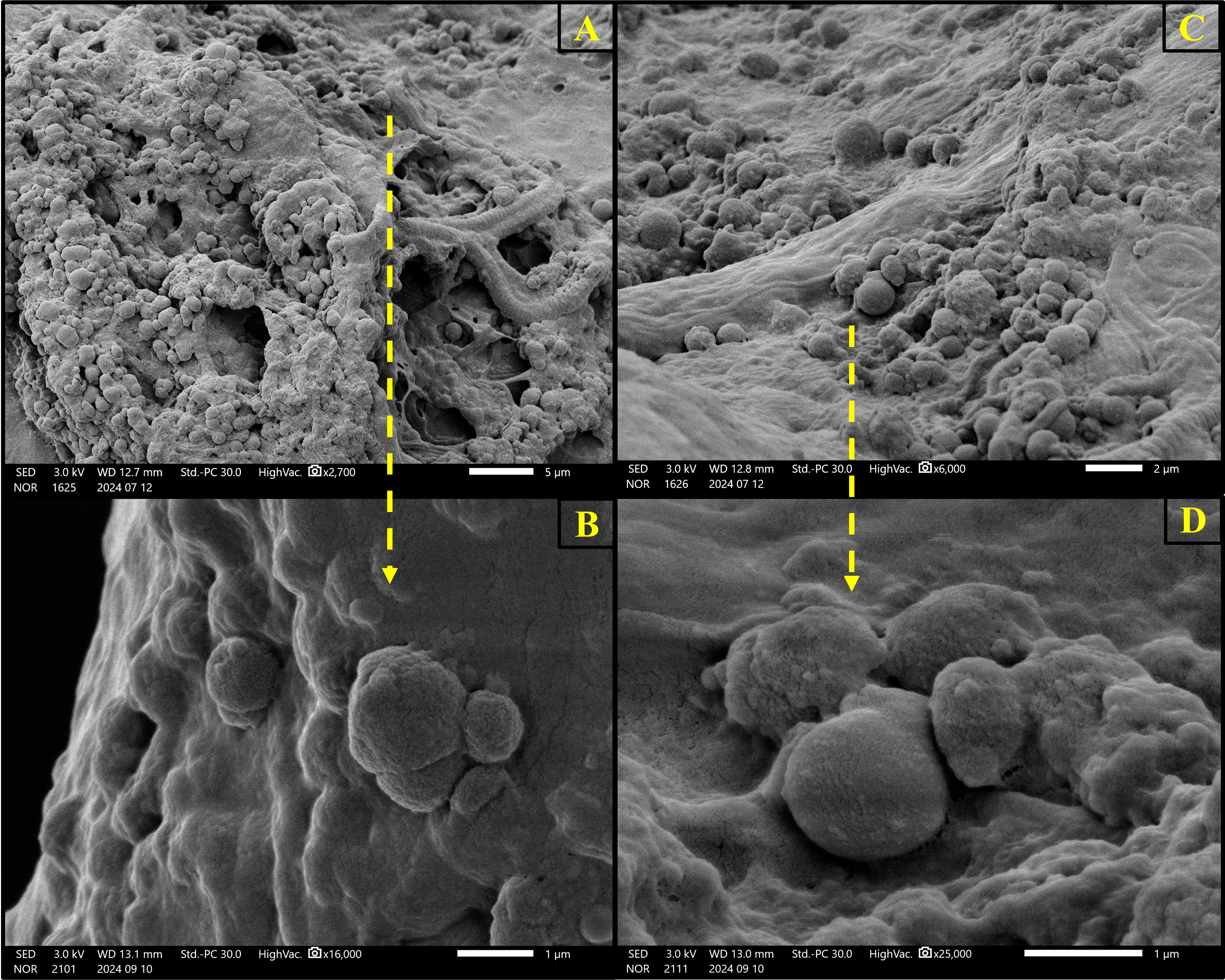

Supplement: Supplementary file 1 [file ijms-27-04526-s001.zip › Figure S5.tif]

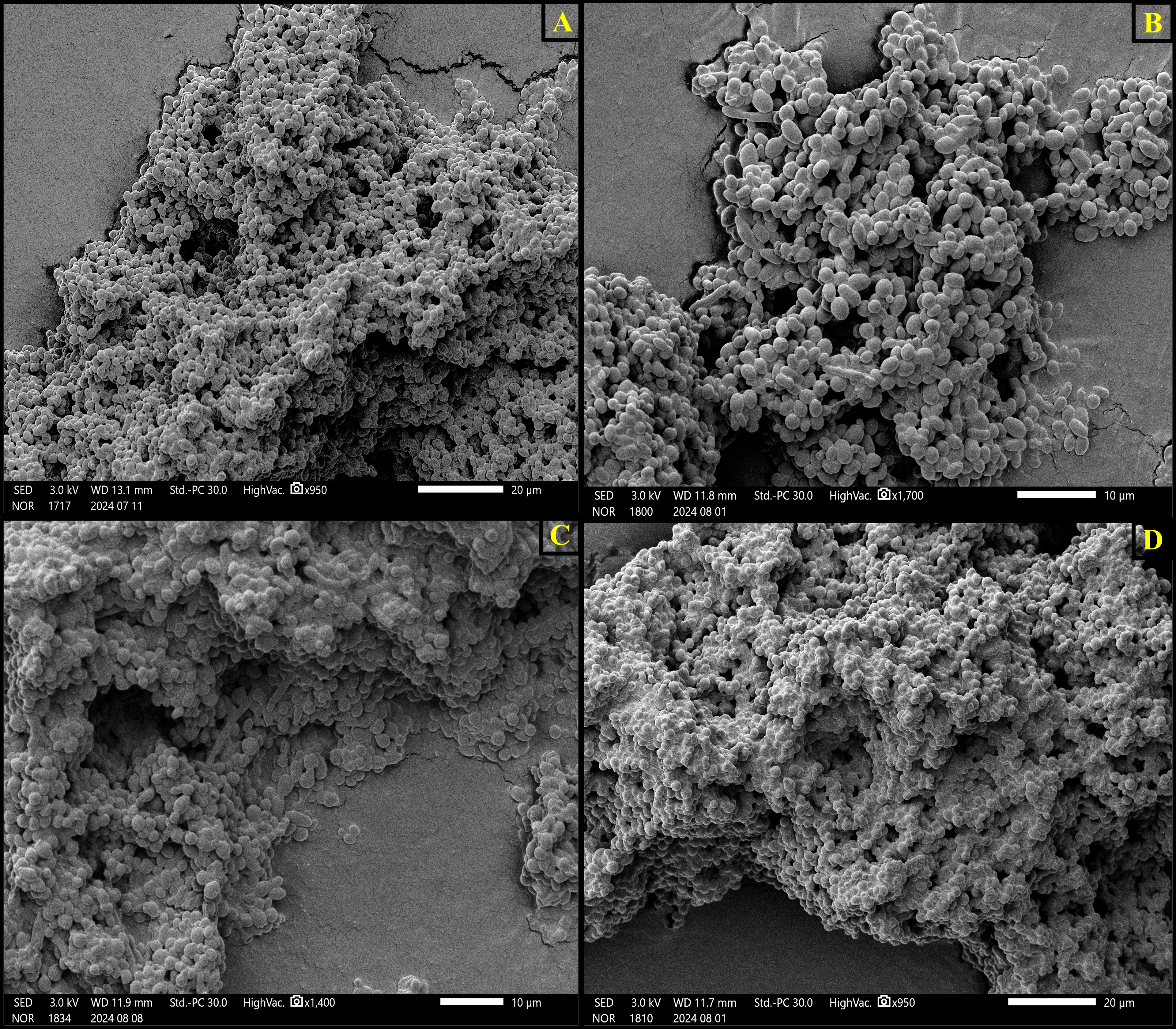

Supplement: Supplementary file 1 [file ijms-27-04526-s001.zip › Figure S6.tif]

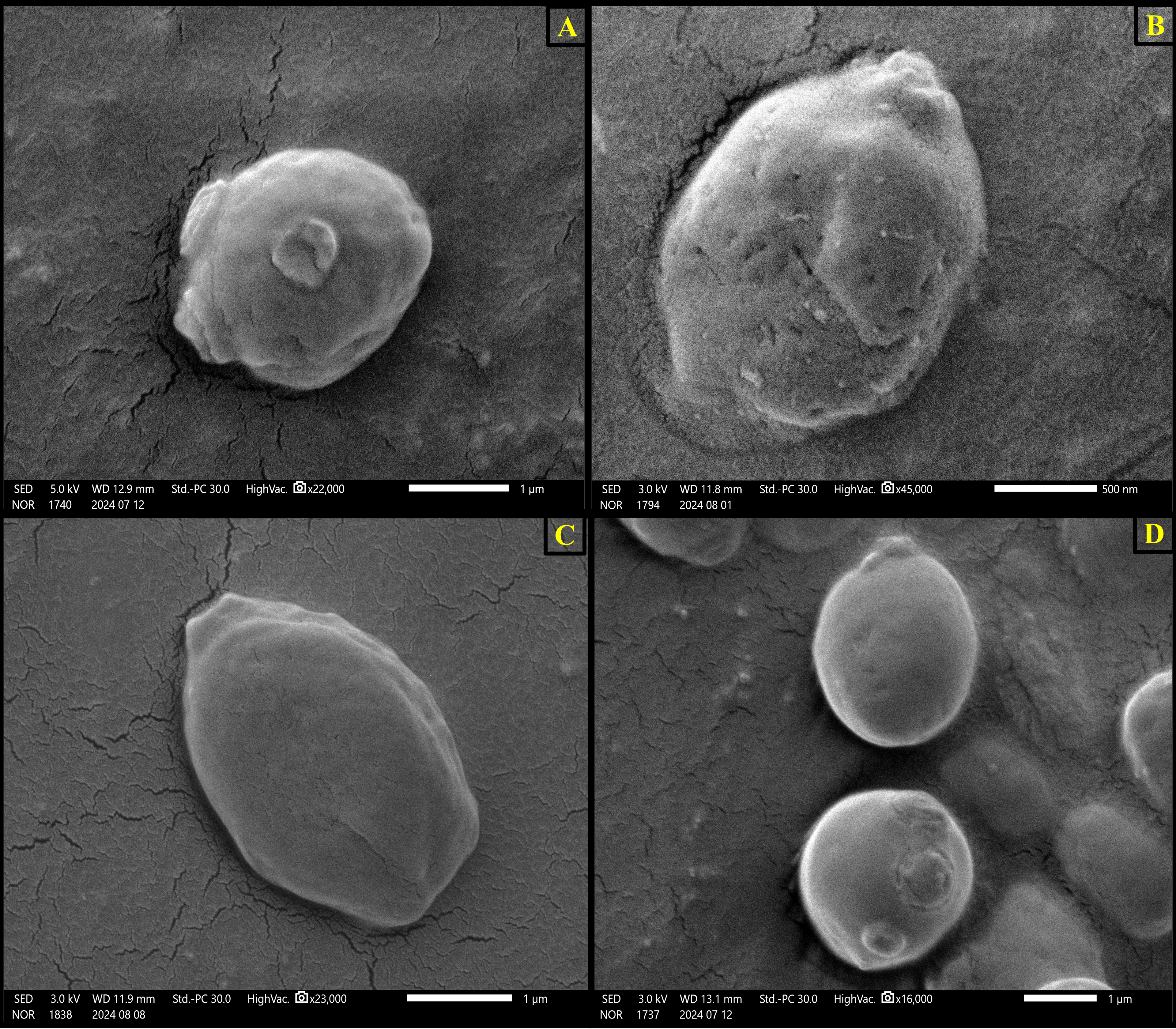

Supplement: Supplementary file 1 [file ijms-27-04526-s001.zip › Figure S7.tif]
